# Insight into the genetics of a novel white-striped leaf in rice

**DOI:** 10.3389/fpls.2025.1622640

**Published:** 2025-08-20

**Authors:** Maiporn Maipoka, Kitti Walayaporn, Wanchana Aesomnuk, Siriphat Ruengphayak, Siwaret Arikit, Apichart Vanavichit

**Affiliations:** ^1^ Rice Science Center, Kasetsart University, Nakhon Pathom, Thailand; ^2^ Department of Agronomy, Faculty of Agriculture at Kamphaeng Saen, Kasetsart University, Nakhon Pathom, Thailand

**Keywords:** white-striped leaf, *Oryza sativa* L., chlorophyll metabolism, chloroplast development, ribonucleotide reductase (RNRS1), SAMHD1, nucleotide metabolism

## Abstract

**Introduction:**

Rice is mainly consumed by half of the world’s population. The imminent climate change and population growth expected in the next 30 years will outpace the current rice production capacity, posing risks to food and nutrition security in developing nations. One simplified approach to address this challenge is to improve photosynthetic capacity by increasing chlorophyll content in leaves and stems.

**Methodology:**

We identified a unique white-striped leaf (*wsl*) mutant, RBR05, which is productive, stage-specific and temperature-sensitive, albeit with low chlorophyll content during the adult stage and recessive to regular solid-green leaf (SGL) rice. We utilised RNA sequencing between the *wsl* and SGL to identify differentially expressed genes (DEGs) and QTL sequencing to identify genes responsible for the *wsl* phenotype.

**Result:**

We identified a single recessive gene controlling *wsl* in RBR05. It is a novel missense mutation (R310H) of *OsSAMHD1*, a key contributor to the *wsl* phenotype in RBR05. The mutation, *wsl310*, turns Arg to His at amino acid position 310 in exon 10, which results in abnormal chloroplast development, a lack of chlorophyll pigment, and the formation of non-chlorophyllous cells in the whitened region of the leaves and leaf sheaths. The *wsl310 (qwsl1_503564)* was associated with decreased gene expression in the formation of photosynthetic machinery and the chlorophyll biosynthetic pathway, while the upregulation of the *OsRNRS1* and genes involved in the expression of plastid-encoded genes was observed. A SNP marker specific for the missense mutation was completely co-segregated with the *wsl310* in the segregating population for *wsl* and SGL, demonstrating that the R310H substitution is responsible for *wsl* in RBR05.

**Discussion:**

Previous reports have shown that *OsSAMHD1* is a hotspot of mutations, which severely affect *wsl* from the seedling to heading stages. In several events, the interaction between *OsRNRS1* and *OsSAMHD1* highlights the critical role of maintaining nucleotide homeostasis and proper chloroplast development in compensating for mutations. The functional marker developed in this study will enable rice breeders to further enhance new leaf colouration and productivity in RBR05.

## 1 Introduction

Rice is a primary source of nutrition for more than half of the global population. According to the United Nations (UN), the global population is expected to reach 9.8 billion by 2050, resulting in increased demand for food and nutrients. One approach is to enhance leaf chlorophyll content and other pigments to improve crop photosynthesis and nutrient density. Leaf plays a significant role in photosynthesis and transpiration. Green leaves accumulate high chlorophyll density, the plants’ most abundant pigments. A few landraces express distinct dark purple leaf blades and sheaths by accumulating a large amount of anthocyanin. Understanding the interplay between leaf chlorophyll and other pigments is one of the strategies for developing new rice varieties. Both pigment accumulation and chloroplast development determine chlorophyll content and leaf colouration.

Generally, rice leaves are green due to the accumulation of chlorophyll, the overall content of which is regulated by both chlorophyll biosynthesis and degradation. Chlorophyll metabolism is strongly linked with the assembly of photosynthetic machinery. Several studies have revealed that disrupting chlorophyll biosynthetic gene expression leads to a reduction in photosynthetic pigment content, aberrant thylakoid structure, altered leaf colouration, and results in a yellow-green leaf phenotype (Zhang et al., 2006, 2015; Liu et al., 2007, 2021a; Wang et al., 2010, 2017b, 2021;Tian et al., 2013; Ruan et al., 2017; Long et al., 2022; Shim et al., 2023; Yao et al., 2023). These key enzymes in the chlorophyll biosynthetic pathway are glutamyl-tRNA synthetase, glutamate-1-semialdehyde aminotransferase, protoporphyrin IX magnesium chelatase, Mg-protoporphyrin IX methyltransferase, and divinyl reductase. Conversely, mutations in chlorophyll degradation genes, such as *chlorophyll b reductase (non-yellow coloring1; OsNYC1)* and *NYC1-like (OsNOL)*, lead to a stay-green phenotype (Kusaba et al., 2007; Sato et al., 2009).

Chloroplasts are semi-autonomous organelles whose development and function are tightly regulated through the coordinated expression of nuclear and plastid genes. Disruptions in genes involved in chloroplast development and function can lead to defective chloroplast ultrastructure, altered pigmentation, and changes in leaf colouration. For example, the loss of function of the plastid sigma factor genes *OsSIG1* and *OsSIG2A*, which encode proteins that facilitate promoter recognition and transcription initiation by PEP (plastid-encoded plastid RNA polymerase), results in pale green and albino leaf phenotypes, respectively (Tozawa et al., 2007; Yu et al., 2019). Furthermore, mutations in genes encoding plastid ribosomal proteins, such as *OsPRPS1/OsASL4*, *OsRPS20/ASL1*, *OsRPL12/OsAL1*, *OsPRPL18*, and *OsRPL21/ASL2*, result in a seedling-lethal albino phenotype (Gong et al., 2013; Lin et al., 2015; Zhao et al., 2016a; Zhou et al., 2021; Chen et al., 2022). Additionally, loss-of-function mutations in genes encoding pentatricopeptide repeat proteins, such as *WSL*, *WSL4*, *WSL5*, and *CDE4*, result in a temperature-sensitive white-striped leaf phenotype (Tan et al., 2014; Wang et al., 2017a; Liu et al., 2018, 2021b). Pentatricopeptide repeat proteins play essential roles in chloroplast post-transcriptional RNA processes. Similar phenotypes have also been observed in mutants with mutations in *multiple organellar RNA editing factor (WSP1)* (Zhang et al., 2017), *HNH endonuclease domain-containing protein (WSL9)* (Zhu et al., 2020), and *PEP-associated protein OsFLN2* (Lv et al., 2017).

In addition, multiple lines of evidence have demonstrated that disruptions in nucleotide metabolism can profoundly affect the balance of the nucleotide pool, impairing essential cellular functions such as DNA/RNA synthesis, DNA damage repair, genome stability, and cell cycle progression. A loss-of-function mutation in *OsPurD*, which encodes an enzyme in the second step of the *de novo* purine biosynthesis pathway, results in a virescent-albino leaf (Zhang et al., 2018). The white-striped leaf phenotype has been observed in the mutants with mutations in *chloroplast adenine nucleotide transporter OsBT1-3* (Hu et al., 2017; Lyu et al., 2017; Zhang et al., 2024), *adenylate kinase 1 (OsAK1)* (Wei et al., 2017), and *nucleoside diphosphate kinase 2 (OsNDPK2)* (Ye et al., 2016; Zhou et al., 2017). The mutation in *OsRNRL1* and *OsRNRS1*, which encode the large and small subunits of ribonucleotide reductase—the rate-limiting enzyme in the *de novo* synthesis of nucleotides (Yoo et al., 2009; Chen et al., 2015; Qin et al., 2017; Shen et al., 2023); *dNK (WSL8)*, which encodes deoxyribonucleoside kinase—the initial enzyme in nucleotide salvage pathway (Liu et al., 2020); and *OsSAMHD1*, which has a proposed role in nucleotide catabolism (Zhao et al., 2016b; Ge et al., 2017; Wang et al., 2022, 2023; Hu et al., 2024), also results in white-striped leaf phenotype.

This study investigated the unique white-striped leaf phenotype in the Rainbow Rice variety, RBR05, which is not lethal, stage-specific, and temperature-sensitive. We employed both QTL-seq and RNA-seq approaches to elucidate the molecular mechanisms and identify a causal gene. Microscopic analysis revealed defective chloroplasts and the presence of non-chlorophyllous mesophyll cells associated with the white-striped pattern in RBR05 leaves. Through QTL-seq analysis, *OsSAMHD1*, which encodes HD domain-containing protein, was identified as a candidate gene responsible for this phenotype. Based on the SNP identified in *OsSAMHD1*, a molecular marker was developed for marker-assisted selection to improve Rainbow Rice varieties.

## 2 Materials and methods

### 2.1 Plant materials and growth conditions

The RBR05 (*wsl*) is one of the five Rainbow Rice varieties obtained by crossbreeding between the white-striped leaf Jao Hom Nin mutant (*wsl*-JHN) and the purple leaf Kum Hom Nin (KHN) provided by Rice Science Center, Kasetsart University, Thailand. The *wsl*-JHN was induced by fast neutron irradiation at a dose of 33 Gy. Riceberry (RB), which exhibits dark green leaves, was included in this study as a control group. Riceberry was developed by crossing the dark green leaf variety Jao Hom Nin (JHN) and the green leaf variety KDML105, provided by the same institute. These plants were grown in a paddy field at the Rice Science Center, Kasetsart University, Thailand.

### 2.2 Determination of pigment content

Pigment content was quantified by using the spectrophotometric method. Chlorophylls (chlorophyll *a*, chlorophyll *b*, and total chlorophyll) were determined according to the previous method (Matsuda et al., 2012). Briefly, the leaf sample was ground into fine powder in liquid nitrogen. Chlorophylls were extracted by incubating the powder in methanol. The absorbance of the mixture was measured at 470, 647, and 663 nm. The pigment content was calculated as described previously (Lichtenthaler, 1987). Total anthocyanin content was determined according to Lao and Giusti (2016) with some modifications. Anthocyanins were extracted from the leaf sample with HCl-acidified methanol. Then, the absorbance of the mixture was measured at 535 nm. The ratio between total anthocyanin and total chlorophyll content was calculated.

### 2.3 Leaf anatomical structure and transmission electron microscopy analysis

The flag leaves from RB and RBR05 at the early booting stage were subjected to leaf anatomical structure and TEM analyses. The leaf samples were freshly sectioned into 30 µm with a Leica VT1000s vibratome and observed with an Olympus BX43 fluorescence microscope equipped with an EP50 camera and a UV wideband filter (U-FUW). For TEM analysis, small pieces were cut from RB and the green and white sectors of RBR05. The samples were fixed in a fixative buffer containing 2.5% glutaraldehyde and 2.5% paraformaldehyde in 0.2 M cacodylate-HCl buffer (pH 7.2). Post-fixation was performed with 1% osmium tetroxide. The samples were then embedded in Spurr’s resin. The ultrathin sections were prepared and subsequently stained with 2% uranyl acetate followed by 0.01% lead citrate. The stained sections were examined using a transmission electron microscope (Hitachi HT7700).

### 2.4 Transcriptome analysis

#### 2.4.1 RNA extraction, library preparation, and sequencing

The youngest fully expanded leaves were collected from RBR05 and RB at 85 days after germination (DAG) for total RNA extraction. Total RNA was isolated using the RNeasy Plant Mini Kit (QIAGEN, USA), following the manufacturer’s protocol with some modifications. The plants were grown in a paddy field at the Rice Science Center, Kasetsart University, Thailand. During the experimental period, the average day/night temperatures were 25°C and 22°C, respectively, with relative humidity ranging from 60% to 70% during the day and 70% to 90% at night. Three biological replicates of the total RNA sample of each rice variety were submitted to the National Omics Center, NSTDA, Thailand, for RNA-seq library preparation and sequencing. The RNA libraries were constructed using the MGIEasy RNA Library Prep Set (MGI Tech, China) and sequenced on the MGISEQ-2000 platform (MGI Tech, China) for paired-end reads of 150 bp.

#### 2.4.2 RNA-seq data analysis

The RNA-seq data analysis was performed using the CLC Genomics Workbench (version 20.0, QIAGEN, USA). The clean reads obtained from the National Omics Center, NSTDA, Thailand, were imported into the CLC genomics workbench (version 20.0, QIAGEN, USA) and mapped to the *Oryza sativa* Nipponbare reference genome (Rice Genome Annotation Project, http://rice.uga.edu/) (Kawahara et al., 2013; Hamilton et al., 2024) with default parameter settings. Differential gene expression analysis was performed using the CLC Genomics Workbench. Genes with log2 fold change ≥ |1| and FDR *p*-value < 0.05 were assigned as differentially expressed.

Gene Ontology (GO) enrichment analysis of differentially expressed genes (DEGs) was carried out using the Singular Enrichment Analysis (SEA) tool in AgriGO V2.0 (https://systemsbiology.cau.edu.cn/agriGOv2/) (Tian et al., 2017). Fisher’s exact test with Hochberg FDR correction was applied, and the minimum number of mapping entries was set at 3. Kyoto Encyclopedia of Genes and Genomes (KEGG) enrichment analysis was conducted using ShinyGO 0.82 (https://bioinformatics.sdstate.edu/go/) (Ge et al., 2020).

### 2.5 QTL-seq methodology and analysis

#### 2.5.1 Construction of the F_2_ population

The F_2_ population was derived from a cross between RBR05 (*wsl*) and PinK+4#78A03 (Solid green leaf, SGL). A total of 232 field-grown F_2_ plants were developed in December 2023 by randomly choosing from the base F_2_ population. The *wsl* phenotype and SPAD were observed in February-March 2024.

#### 2.5.2 Establishment of distinctive phenotypic groups

To find genes affecting *wsl*, two groups of 30 individuals each were sequenced: one clearly expressing the *wsl* and the other expressing SGL. The leaf colour patterns were visualised at 90 and 110 days after germination (DAG). The chlorophyll contents were measured in three positions on the penultimate leaves using the chlorophyll meter SPAD device. The chlorophyll contents were measured twice at 90 (SPAD1) and 110 DAG (SPAD2) from February to March 2024.

#### 2.5.3 Genomic DNA for sequencing

High-quality genomic DNA was isolated from young leaves of the parents (PinK+4#78A03 and RBR05) and 60 individual lines (30 *wsl* and 30 SGL lines) selected from 232 F_2_ individuals using the DNeasy Plant Mini Kit (QIAGEN, Hilden, Germany) and monitored using a Nanodrop spectrophotometer and agarose gel electrophoresis, respectively, for whole-genome sequencing (WGS).

#### 2.5.4 QTL-seq analysis and candidate gene annotation

Whole-genome sequencing of individual lines was analysed using the MGI-seq platform at China National Genebank (CNGB; Shenzhen, China). The raw sequencing data were filtered and processed using Trimmonatic software version 0.30 (Bolger et al., 2014) to obtain the clean data using QTL-seq pipeline v2.2.2 (http://github.com/YuSugihara/QTL-seq). The genomic sequence of the RBR05 parent was used as a reference genome for mapping reads of the two bulk samples. The generated reference genome, referred to as the RBR05-based pseudo-reference genome, was developed by aligning RBR05 clean reads to the public reference genome, Nipponbare IRGSP1.0, and substituting the genome of Nipponbare with the variants of RBR05 parent. The clean reads of each F2 sample were in equal numbers and pooled in SGL- and *wsl*-bulks. SNP calling and SNP index were calculated using the QTL-seq pipeline (Takagi et al., 2013). SNP index of SGL- and *wsl*-bulks was computed from the ratio between the SNP alleles of RBR05 and the total number of reads corresponding to the SNP. The ΔSNP index was calculated according to the formula:


Δ(SNP index) = [(SNP index of SGL−bulk) − (SNP index of wsl−bulk)]

A sliding window analysis was performed by averaging the ∆ (SNP index) with a window size of 1 Mb, with 250 kb steps. The minimum aligned read depth cutoff for obtaining SNPs was set to eight reads. The candidate genes within the detected QTL regions were obtained from the Rice Genome Annotation Project (https://rice.uga.edu/index.shtml) (Kawahara et al., 2013; Hamilton et al., 2024). Only annotated genes were considered candidates for further filtering to obtain those containing SNPs/Indels with moderate or high effects. The SNP effects were determined using SnpEff & SnpSift (https://pcingola.github.io/SnpEff/).

### 2.6 Functional marker development

A missense-variant (CGT/CAT) of *LOC_Os01g01920* on chromosome 1 at the 929-nucleotide sequence on exon 10 was identified as the functional SNP for *wsl*. A functional marker was developed based on KASP (Kompetitive Allele Specific PCR).

#### 2.6.1 Validation of functional marker

The validation population was an F_4_ population derived from RBR05 (*wsl*) x PinK+6#3E01 (SGL). This SGL parent was different from the QTL-seq population, RBR05 x PinK+4 #78A03, to confirm its accuracy. A total of 211 individual plants were grown from January to April 2025 at the Rice Science Center, Kasetsart University, Thailand. *wsl* phenotyping was conducted after flowering. Genomic DNA from individual plants was isolated for functional marker analysis.

## 3 Results

### 3.1 Phonotypic characterisation of the white-striped leaf RBR05

**Figure 1:**
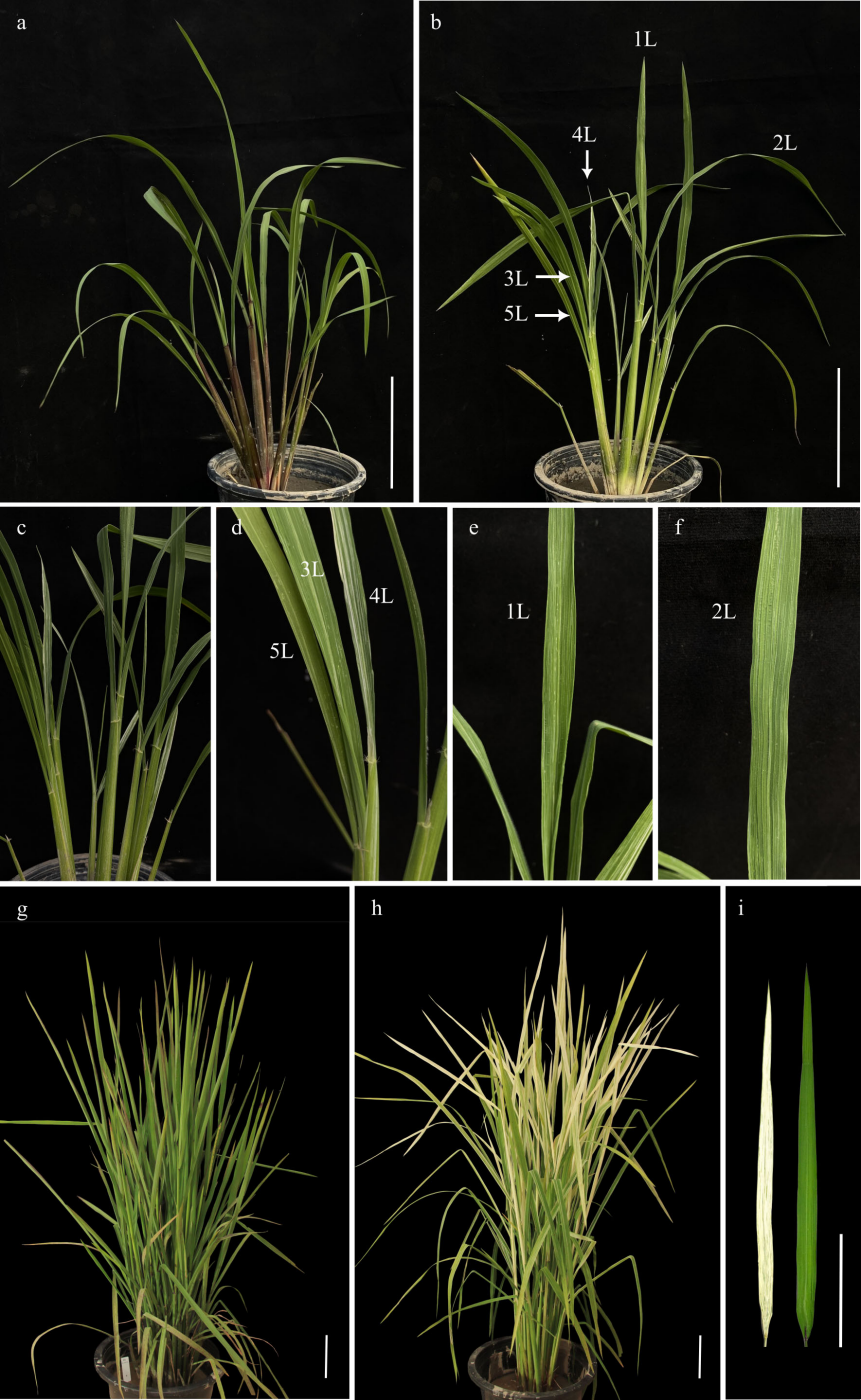
Phenotypes of solid green leaf Riceberry and white-striped leaf Rainbow rice 05 (RBR05). **(a)** Riceberry and **(b)** RBR05 at the tillering stage (40-day-old plants). **(c-f)** Enlarged leaves of **(b)**. **(d)** Lateral stem leaves (3L = youngest leaf, 4L = youngest fully expanded leaf, 5L = second fully expanded leaf). **(e)** Youngest leaf on the main culm (1L). **(f)** Youngest fully expanded leaf on the main culm (2L). **(g)** Riceberry and **(h)** RBR05 at the early booting stage. **(i)** Flag leaf of Riceberry (right) and RBR05 (left) at the early booting stage. Scale bars = 10 cm.

**Figure 2:**
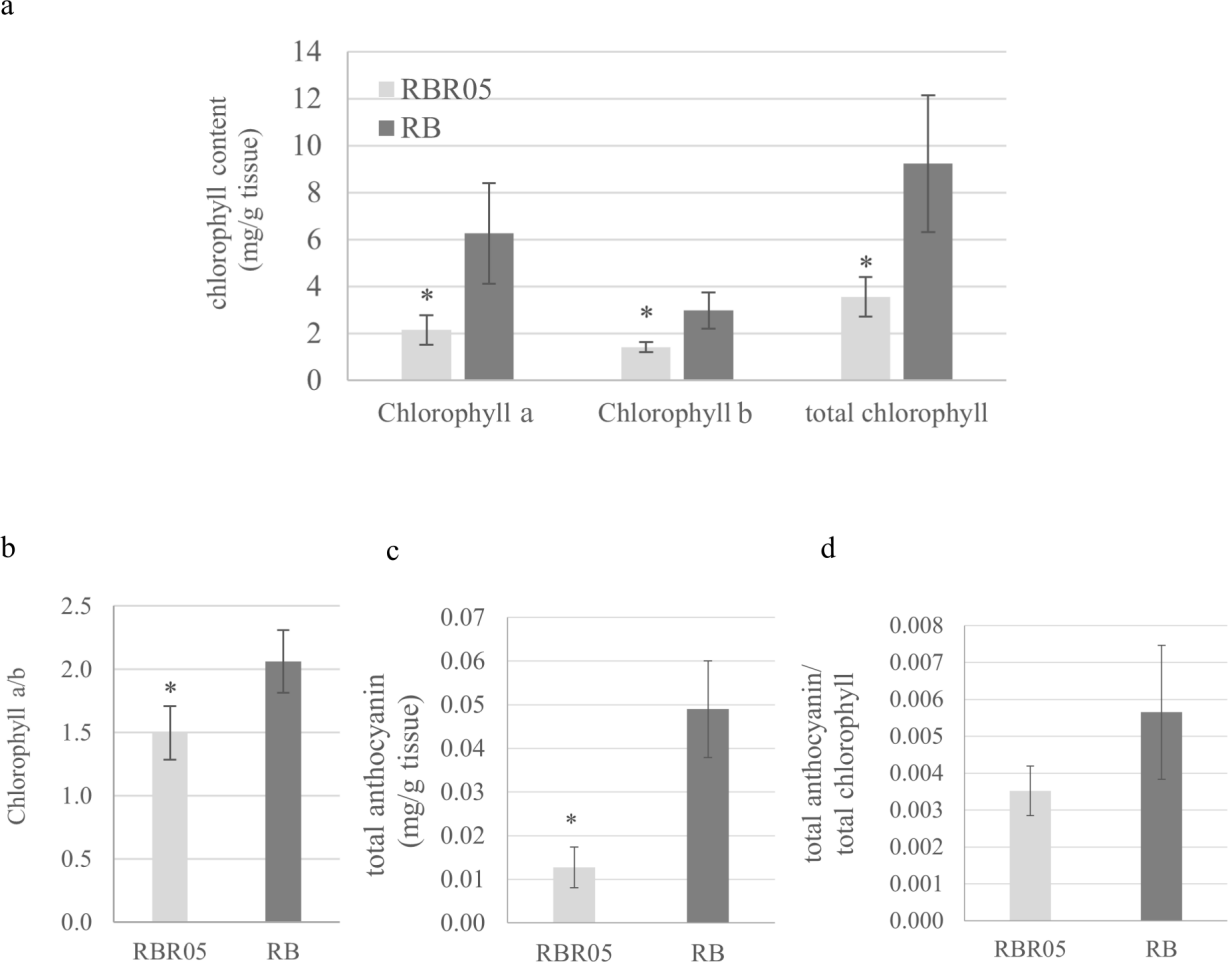
Pigment content in Riceberry and RBR05 flag leaves. **(a)** chlorophyll a, chlorophyll b, and total chlorophyll content. **(b)** chlorophyll a/b ratio. **(c)** total anthocyanin content. **(d)** total anthocyanin to total chlorophyll ratio. Data are presented as mean ± SD (n = 4) (t-test; * *p* < 0.05).

RBR05 exhibited a white-striped leaf in a growth stage- and temperature-dependent manner (**Figure 1**, **Supplementary Figure 1**). It germinated normally like a wild-type plant. Under paddy field conditions, RBR05 exhibited green leaves during the seedling stage. It gradually developed longitudinal white stripes on the new green leaves, starting at the beginning of the tillering stage and continuing until maturity. At the early tillering stage, the newly emerging leaves from the lateral culm displayed more prominent variegation than those on the main culm. The flag leaf showed an obvious variegated phenotype, characterised by predominantly albino areas with few narrow green stripes, depending on the temperature below 25°C. Consistent with the visible phenotype, the chlorophyll content in RBR05 decreased as the proportion of white-striped leaf area increased (**Figure 2**).

**Figure 3:**
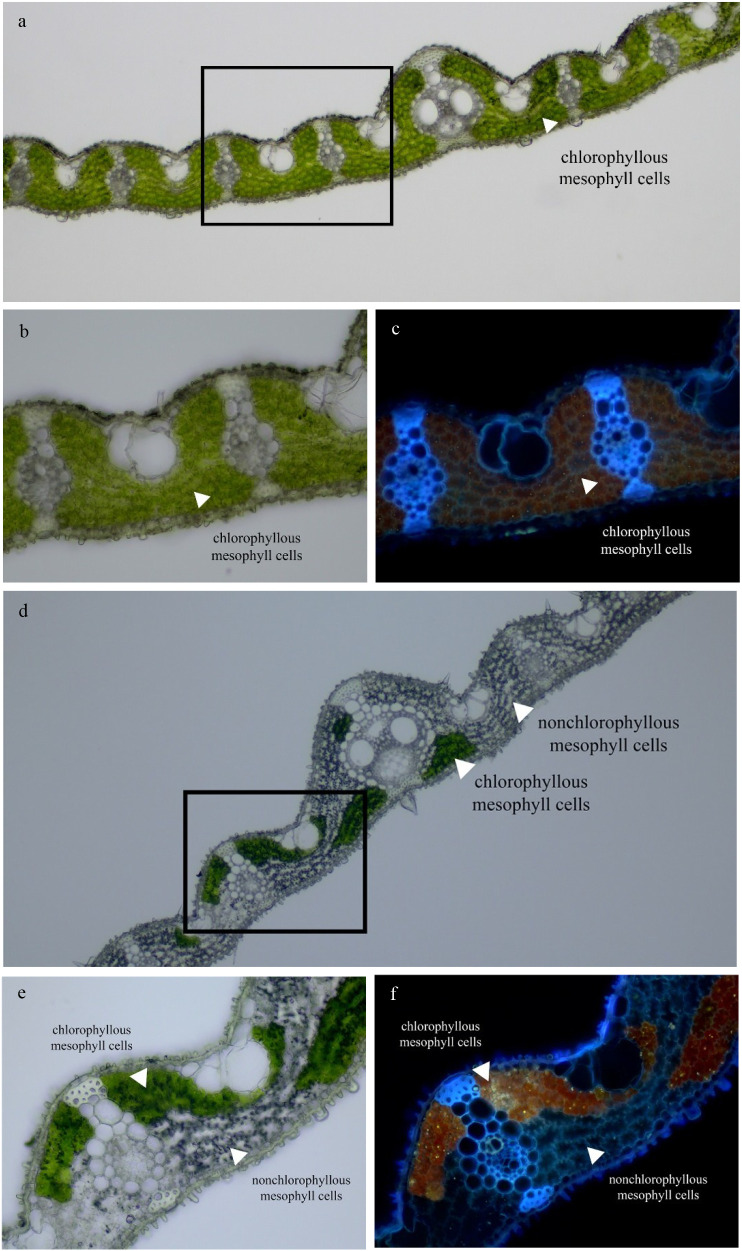
Flag leaf anatomical structure of Riceberry and RBR05. Transverse section of flag leaf of Riceberry **(a–c)** and RBR05 **(d–f)** under bright field light microscope **(a, b, d, e)** and under fluorescence microscope equipped with UV filter wideband (U-FUW) **(c, f)**.

**Figure 4:**
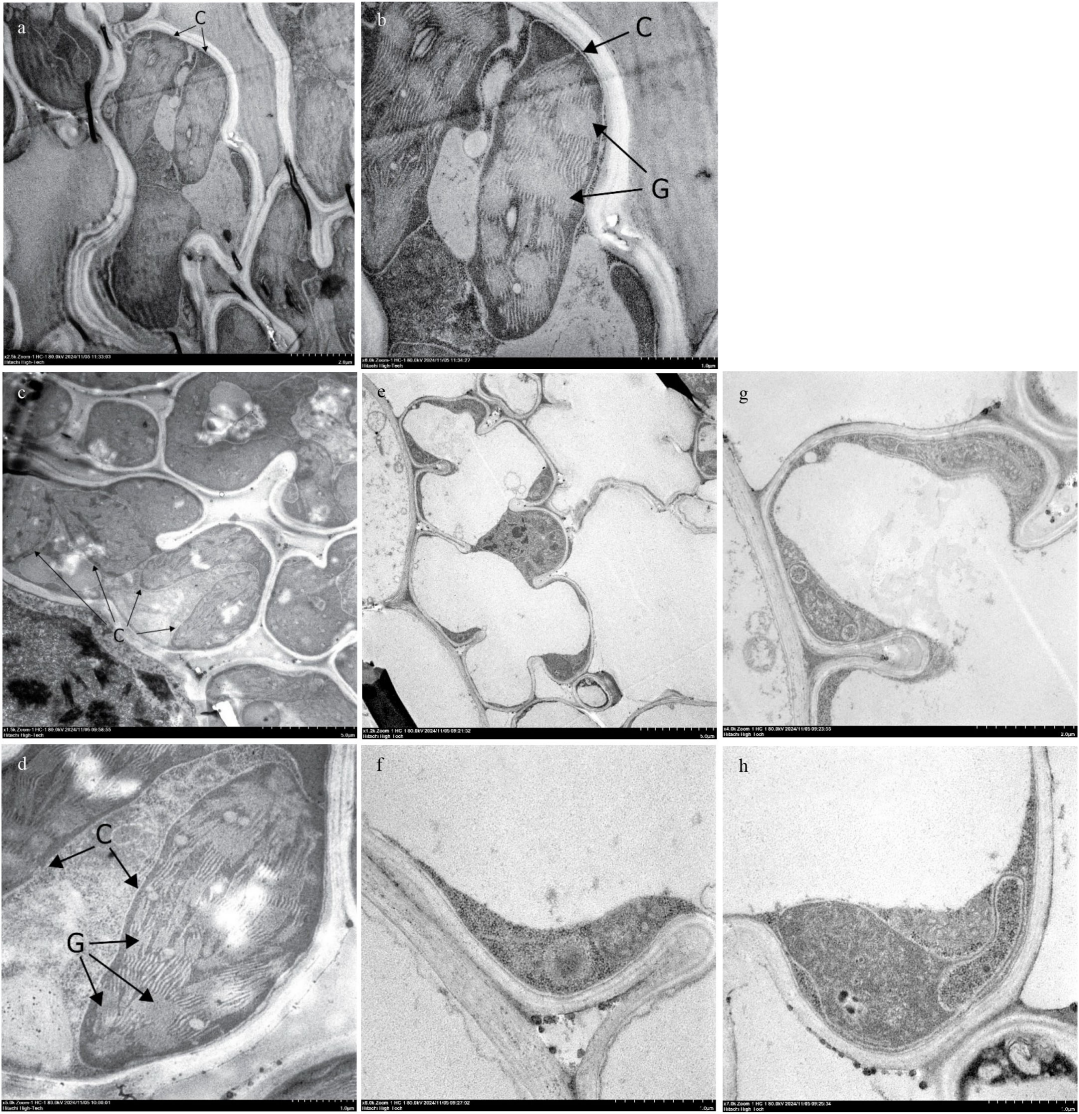
Transmission electron microscope (TEM) images of chloroplast of Riceberry and RBR05. The ultrastructure of chloroplasts in the mesophyll cell of the flag leaf of Riceberry **(a, b)**, green leaf sector **(c, d)** and white leaf sector **(e–h)** of RBR05. C, chloroplast; G, grana.

The transverse section of the RBR05 flag leaf, observed under a bright-field microscope and a fluorescence microscope equipped with a UV wideband filter (U-FUW), revealed a random distribution of chlorophyllous and non-chlorophyllous mesophyll cells (**Figure 3**). Non-chlorophyllous mesophyll cells were characterised by their transparent appearance under the bright-field microscope and the absence of red chlorophyll autofluorescence under the fluorescence microscope. Transmission electron microscopy (TEM) was employed to examine whether the white stripes and non-chlorophyllous mesophyll cells were associated with defective chloroplast development. In Riceberry (RB) and the green sector of RBR05, mesophyll cells contained abundant chloroplasts with well-organised ultrastructure. A highly developed thylakoid membrane system was observed. Conversely, mesophyll cells in the white sector of RBR05 were dominated by large empty spaces with chloroplast-like structures presenting as flattened shapes adhered to the cell wall (**Figure 4**).

### 3.2 Transcriptome analysis

The RNA-seq approach was used to explore the molecular basis underlying the white-striped leaf (*wsl*) phenotype observed in RBR05. Differentially expressed genes (DEGs) between *wsl* RBR05 and the solid dark-green leaf (SGL) RB were identified using such criteria as log2 fold change ≥ |1| and FDR *p*-value < 0.05. 94 DEGs were identified, including 78 upregulated and 16 downregulated in RBR05 (**Supplementary Table 1**). To investigate the biological functions of DEGs in RBR05, Gene Ontology (GO) and Kyoto Encyclopedia of Genes and Genomes (KEGG) enrichment analyses were initially performed. Due to the small number of DEGs, the stringency of enrichment settings was adjusted, as described in the Materials and methods section. GO analysis identified significant overrepresentation of terms related to photosynthesis (light reaction) and oxidation-reduction processes (**Supplementary Figure 3**). Within the GO term oxidation reduction, *protochlorophyllide reductase A (OsPORA)*, a key gene in chlorophyll biosynthetic pathway, was identified (**Supplementary Table 2**). KEGG analysis identified flavonoid biosynthesis as the only significantly enriched pathway (FDR < 0.05). Although additional pathways, such as photosynthesis-antenna protein and porphyrin metabolism, were also detected, they did not meet the significance threshold (**Supplementary Figure 3**; **Supplementary Table 3**).

To gain a more comprehensive view, we manually classified DEGs based on sequence homology and literature-based functional annotation (**Supplementary Table 1**). A number of these DEGs are related to chloroplast biogenesis and photosynthesis (**Table 1**). For instance, genes associated with the photosynthetic apparatus were downregulated in RBR05, including *chlorophyll A-B binding protein OsLhcb1.1*, which encodes a component of the PSII light-harvesting (antenna) complex; *cytochrome b6-f complex subunit IV*; and *a homolog of AtPsaP/AtCURT1B*, which encodes a member of the Curvature Thylakoid1 (CURT1) protein family. Conversely, *the photosynthetic reaction centre protein OsPsbA/D1* was upregulated in RBR05.

**Table 1:** Differentially expressed genes (DEGs) between RBR05 (white-striped leaf mutant) and Riceberry related to chloroplast development and photosynthesis.

Gene ID	Description	Riceberry vs. RBR05 – Log fold change	FDR p-value
Nucleotide metabolism			
*LOC_Os06g14620*	*ribonucleoside-diphosphate reductase small chain (OsRNRS1)*	-1.78	0.01
Chloroplast gene expression			
*LOC_Os03g40550*	*kinase, pfkB family (OsFLN2)*	-1.87	0.04
*LOC_Os05g47850*	*chloroplastic group IIA intron* sp*licing facilitator CRS1*	-1.63	0.04
*LOC_Os09g30466*	*nuclear ribonuclease Z (OsTRZ2)*	-1.83	0.03
*LOC_Os05g22724*	*chloroplast 50S ribosomal protein L16*	-6.27	0.00
Chloroplast division			
*LOC_Os12g07880*	*dynamin family protein (OsDRP5B)*	-2.51	0.01
Photosynthetic apparatus			
*LOC_Os01g52240*	*chlorophyll A-B binding protein (OsLhcb1.1)*	6.18	0.00
*LOC_Os08g35420*	*photosynthetic reaction centre protein (OsPsbA/D1)*	-4.64	0.00
*LOC_Os10g39150*	*expressed protein (ortholog of AtPsaP/AtCURT1B)*	2.37	0.00
*LOC_Os01g57945*	*cytochrome b6-f complex subunit 4*	1.68	0.00
Chlorophyll biosynthesis			
*LOC_Os04g58200*	*protochlorophyllide reductase A (OsPORA)*	3.38	0.01
Photorespiration			
*LOC_Os01g32830*	*expressed protein (OsPLGG1)*	2.22	0.00

Furthermore, RBR05 showed increased expression of genes involved in chloroplast gene expression. These genes included *OsFLN2*, which is the rice homolog of *AtFLN2* (a component of plastid transcriptionally active chromosome pTAC); *Chloroplastic group IIA intron* sp*licing facilitator CRS1*; *tRNA 3′-end processing enzyme OsTRZ2*; and *Chloroplast 50S ribosomal protein L16*. *OsDRP5B/ARC5 (Dynamin-Related Protein 5B;* also known as *Accumulation and Replication of Chloroplasts 5*), which encodes a dynamin-related protein GTPase, was upregulated in RBR05. In alignment with the reduction of chlorophyll content, *OsPORA*, one of the two *OsPOR* genes (*OsPORA* and *OsPORB*) in the rice genome, was downregulated in RBR05. Protochlorophyllide reductase (POR) catalyses the reduction of protochlorophyllide *a* (Pchlide *a*) to chlorophyllide *a* (chlide *a*), a light-dependent reaction in the chlorophyll biosynthesis pathway. RBR05 also demonstrated the downregulation of *OsPLGG1a*, which encodes one of the two isoforms of plastidic glycolate/glycerate translocator 1 (OsPLGG1) in rice. PLGG1 is a key transporter in the photorespiratory pathway, facilitating the transport of glycolate and glycerate between chloroplasts and peroxisomes. Interestingly, we detected significant upregulation of *OsRNRS1*, which encodes the small subunit of ribonucleotide reductase (RNR) in RBR05 white-striped leaves. *OsRNRS1* was also associated with the GO term oxidation-reduction (**Supplementary Table 2**).

### 3.3 Developing *wsl* distinctive QTL F_2_ population

The F_2_ plants (RBR05 x PinK+4#78A03) were selected for white-striped and solid green lines, with 30 lines per group, by visual assessment and chlorophyll content measurements using a SPAD meter. For SPAD measurement, two measurements were made to observe the change in chlorophyll content in the leaves. SPAD values were measured in the SGL- and *wsl*-bulks at 90 days after germination (DAG; SPAD1) and 110 DAG (SPAD2). At 90 DAG, the SGL- and *wsl*-bulks showed average SPAD values of 43.2 and 28.5, respectively. For 110 DAG, the SGL- and *wsl*-bulks showed average values of 49.3 and 26.2, respectively (**Supplementary Figure 5** and **Supplementary Table 4**).

### 3.4 White-striped leaf is recessive to solid-green leaf

The 232 F_2_ individuals were derived from a cross between PinK+4#78A03 (SGL) and RBR05 (*wsl*). The *wsl* line showed white-striped leaves in the tillering stage (starting at 80 DAG) accounted for 60 of 232 individuals, while the remaining 172 of 232 individuals observed solid green leaf phenotypes. All F_1_ plants exhibited SGL phenotypes similar to PinK+4 #78A03; therefore, the F_2_ segregation pattern fitted a 3:1 SGL: *wsl* phenotypic ratio (**Supplementary Table 5**). It indicated that a single recessive nuclear gene controlled the white-striped phenotype.

### 3.5 Whole genome sequencing and QTL analysis

As a result, 150-bp clean paired-end sequences were generated. In total, approximately 2,009.62 million reads for *wsl*-bulk, 1,692.91 million reads for SGL-bulk, 54.19 million reads for PinK+4#78A03, and 55.75 million reads for RBR05 were generated, which were equivalent to 292.55, 245.35, 7.85, and 8.09 Gb for the *wsl*-bulk, the SGL-bulk, PinK+4#78A03, and RBR05, respectively (**Supplementary Table 6**). The average sequencing depths for *wsl*-bulk, SGL-bulk, PinK+4#78A03, and RBR05 were 22.85, 19.5, 18.8, and 19.2, respectively. The alignment of the reads from two bulks and parents to the reference genome of Nipponbare revealed 90.27%,91.32%, 91.76%, and 91.03% of read alignments in *wsl*-bulk, SGL-bulk, PinK+4#78A03, and RBR05, respectively, corresponding to 92.71%, 92.46%, 91.73%, and 92.05% of rice genome coverage (**Supplementary Table 6**)

**Figure 5:**
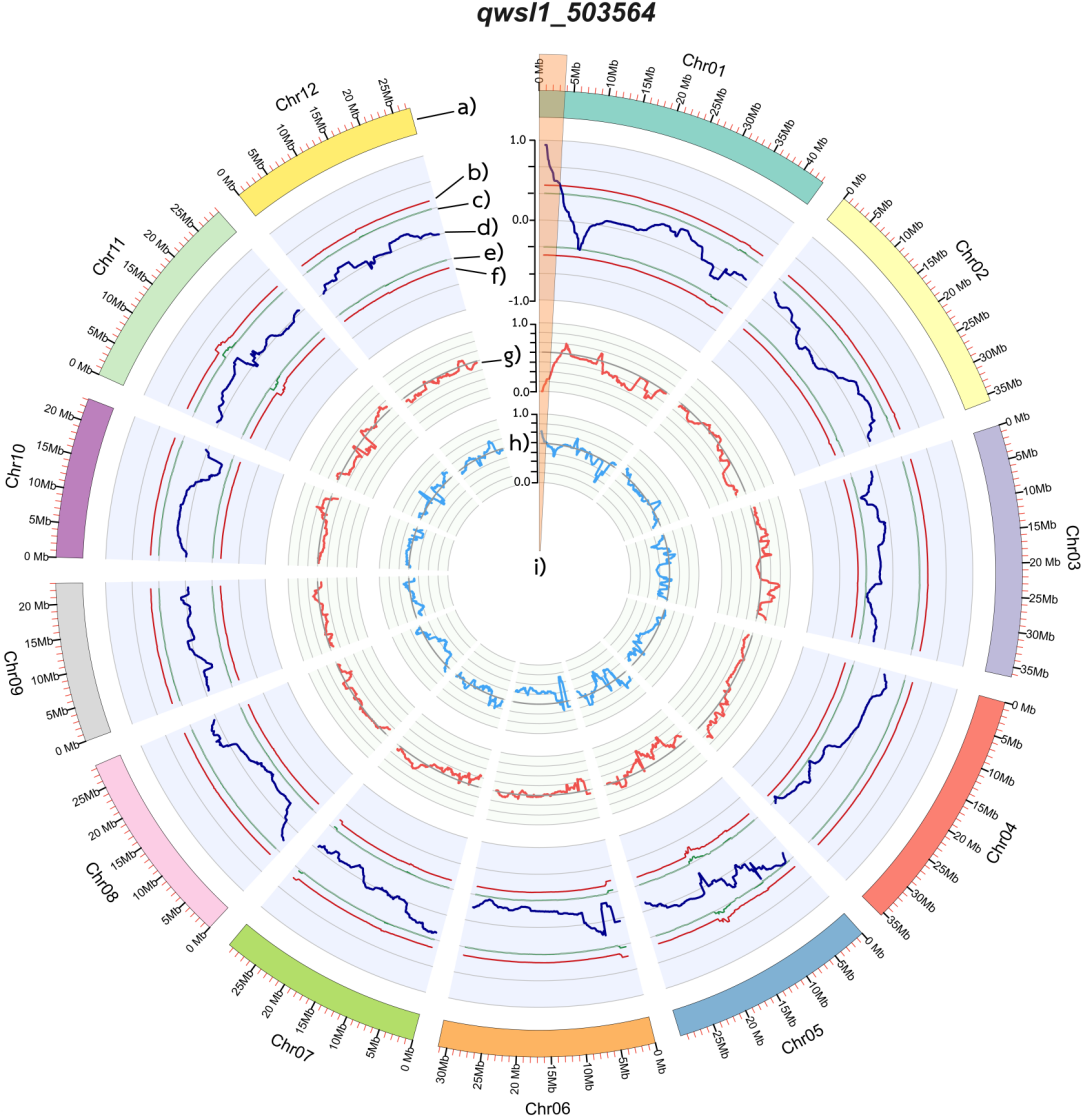
Diagrams showing the single nucleotide polymorphism [SNP] for two bulks (SGL-bulk and *wsl*-bulk) and a comparison of the Δ(SNP index) between them. **(a)** Pseudomolecules of the Nipponbare reference genome (IRGSP 1.0). **(b)** Upper probability values at the 99% confidence level (*p* < 0.01). **(c)** Upper probability values at the 95% confidence level (*p* < 0.05). **(d)** Sliding window plots of Δ(SNP index). **(e)** Lower probability values at the 95% confidence level (*p* < 0.05). **(f)** Lower probability values at the 99% confidence level (*p* < 0.01). **(g)** Sliding window plots of the average SNP index values in the *wsl*-bulk. **(h)** Sliding window plots of average SNP index values in the SGL-bulk. **(i)** Candidate genomic regions containing QTLs for the white-striped leaf.

QTL-seq analysis utilised common SNP variants identified in both the *wsl-* and SGL-bulks, as determined by read mapping against the RBR05 parental genome. Initially, 132,950 SNPs were identified in the two bulks (**Supplementary Table 7**), which required a read-support criterion of at least eight reads. For identifying significant QTLs, the ΔSNP index plot illustrated the differences between the SNP indexes of the SGL-bulk and the *wsl*-bulk. The SNP indexes in each bulk and the ∆SNP indexes were physically plotted across all rice chromosomes (**Figure 5**). We mapped the putative *qwsl1_503564* QTL to the 3.7 Mb region between 0.03 and 3.7 Mb on the short arm of chromosome 1, in which the ΔSNP index was greater than the confidence threshold intervals at 99% (**Figure 5**).

### 3.6 OsSAMHD1 is responsible for the white-striped leaf in RBR05

**Figure 6:**
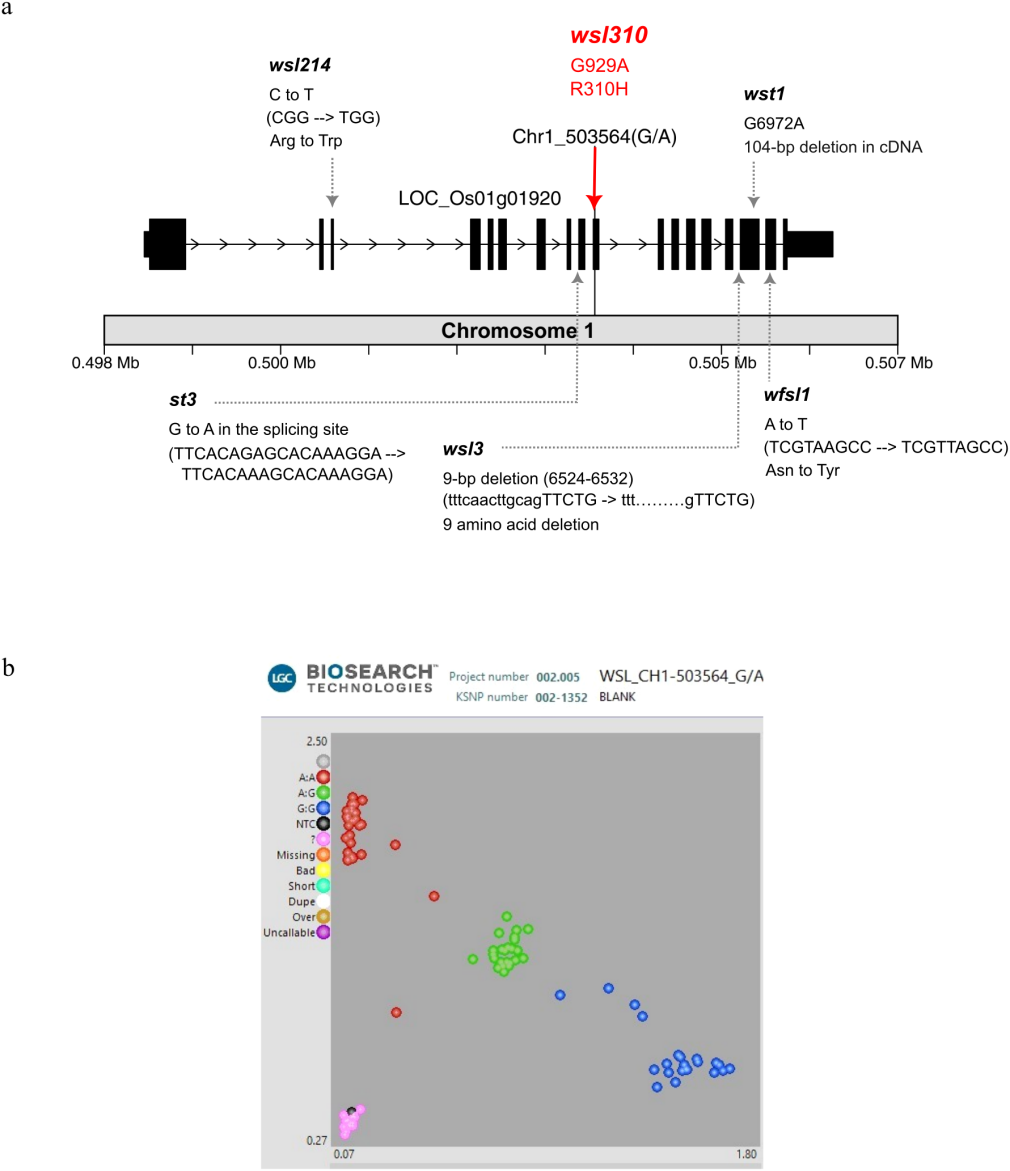
Structure of the *LOC_Os01g01920* gene and KASP genotyping of F_4_ progeny. **(a)** *LOC_Os01g01920* gene contains 18 exons and is located on chromosome 1. A red solid line indicates the nucleotide position 929, where a missense variant (CGT→CAT), named *wsl310*, occurs in exon 10, resulting in an arginine-to-histidine (R310H) amino acid substitution. Other allelic mutation sites have been reported previously, including *wsl3* (Zhao et al., 2016b), *wfsl1* (Ge et al., 2017), *st3* (Wang et al., 2022), *wsl214* (Wang et al., 2023), and *wst1* (Hu et al., 2024). **(b)** KASP genotyping in the F_4_ population derived from a cross between RBR05 (*wsl*) and Pink+6#3E01 (SGL). The red dot represents the A/A allele, which indicates the white-striped leaf (*wsl*) phenotype. The blue and green dots represent the G/G and G/A alleles, respectively, both of which exhibit the normal solid green leaf phenotype.

The *qwsl1*_503564 is located within a genomic region containing 186 mutation sites from 104 annotated genes. Candidate genes with ΔSNP index confidence intervals greater than 95% are listed in **Supplementary Table 8**. Of these, the gene *LOC_Os01g01920* showed the most significant ΔSNP index within the region. This gene encodes the protein called OsSAMHD1, a metal-dependent phosphohydrolase containing HD domain, a homolog of the human dNTPase SAMHD1 and the *Arabidopsis* VEN4. Research indicates that SAMHD1 is crucial for dNTP catabolism in plants and mammals. SAMHD1 plays an essential role in chloroplast biogenesis in plants, including rice and *Arabidopsis*. We precisely identified a functional SNP located at nucleotide position 929 in exon 10, causing a G-to-A missense variant (CGT/CAT) that leads to Arg-to-His amino acid change at residue 310 (**Figure 6**). More significantly, we can precisely differentiate *wsl* from SGL plants at this locus using the functional marker developed from the A-G SNP (**Figure 6**). Therefore, we conclude that the *wsl310* mutation is responsible for the low-temperature-dependent white striped leaf phenotype in RBR05.

## 4 Discussion

### 4.1 Allelic mutations in *OsSAMHD1* affecting the white-striped leaf

The current mutation, named *wsl310*, observed in RBR05, is located in *OsSAMHD1*. The expression of the white-striped leaf phenotype is stage-dependent and responsive to low temperatures. Several studies have demonstrated that mutations in the *OsSAMHD1* gene lead to white-striped leaf phenotypes. Allelic mutations in *OsSAMHD1* have been previously reported at five different mutagenic sites (**Figure 6**). First, the *wsl3* mutant displayed a visible white-striped leaf in both young seedlings and flag leaves of mature plants (Zhao et al., 2016b). The *wsl3* mutant carries a 9-bp deletion (caacttgca, residues 6524-6532) at the junction between intron 15 and exon 16, resulting in a 27-nucleotide deletion (TTCTGCAATGAATATTCTGTTCCAAAG, residues 1300-1326) in the *wsl3* cDNA, causing a 9-amino acid deletion. The wild-type *WSL3* is *OsSAMHD1*, an HD domain-containing protein involved in chloroplast biosynthesis and development in rice (Zhao et al., 2016b).

Second, the *wfsl1* mutant, exhibiting a white, fine-striped leaf phenotype from the tillering stage and an abnormal chloroplast structure, contains a non-synonymous mutation (A>T) in exon 17, causing the amino acid change from Asn to Tyr (Ge et al., 2017). In the wild-type, WFSL1, *OsSAMHD1* is highly expressed in mature leaves and leaf sheaths, and it was localised to the chloroplasts (Ge et al., 2017).

Third, the *wsl214* mutant exhibited white-striped leaves, defective chloroplast development, reduced net photosynthetic rate, and overexcitation of the photosynthetically active reaction center (Wang et al., 2023). The *wsl214* mutant carries a non-synonymous mutation in exon 3, resulting in an amino acid change from Arg to Trp (Wang et al., 2023). In contrast, the wild-type *WSL214*, which encodes an HD domain phosphohydrolase, was widely expressed in various rice tissues, with the highest level observed in leaf tissue. *WSL214* promoted the homeostasis of rice leaf cellular ROS, the reactive oxygen species, by increasing the expression of the catalase gene *OsCATC* and promoting chloroplast development.

Fourth, the *wst1* mutant harbors a splice-site variant at nucleotide position 6972 (G>A), resulting in a 104 bp deletion in the cDNA and impairing the thylakoid membrane structure in the chloroplast (Hu et al., 2024).

Fifth, the *STRIPE3 (st3)* mutant exhibited white-striped leaves with reduced chlorophyll content and abnormal chloroplast development during the seedling stage, but gradually produced nearly normal green leaves as it developed (Wang et al., 2022). A single base mutation (G to A) was identified at the splicing junction in the eighth intron, resulting in abnormal splicing of the transcript. The wild-type *ST3* encodes a human deoxynucleoside triphosphate triphosphohydrolase (dNTPase) in the SAMHD1 homolog (Wang et al., 2022). The wild-type *ST3* was highly expressed in the third leaf at the three-leaf stage and expressed constitutively in the root, stem, leaf, sheath, and panicle, and the encoded protein, OsSAMHD1, was localised to the cytoplasm.

Therefore, *OsSAMHD1* plays critical roles in chloroplast development from the seedling to the flag-leaf stage, regulating nucleotide pools for chloroplasts and maintaining homeostasis of leaf cellular ROS.

### 4.2 Putative functions of OsSAMHD1 variants

A cell maintains nucleotide homeostasis by regulating nucleotide *de novo* synthesis, nucleotide salvage, and nucleotide catabolism. Using QTL-seq analysis in this study, we identified a missense mutation at R310 (R310H) within the conserved domain of OsSAMHD1 as potentially responsible for the white-striped leaf phenotype in RBR05. *OsSAMHD1* gene encodes a metal-dependent phosphohydrolase with an HD domain, a homolog of the human SAMHD1 and *Arabidopsis* VEN4. Unlike human SAMHD1, which contains both an N-terminal SAM domain and an HD domain, OsSAMHD1 and *Arabidopsis* VEN4 have only an HD domain (Sarmiento-Mañús et al., 2025). This conserved HD domain is essential for dNTPase activity. Human SAMHD1 forms a tetramer and functions in nucleotide catabolism and damage repair (Morris and Taylor, 2019; Coggins et al., 2020). It converts 2’-deoxynucleotide-5’-triphosphate (dNTPs) into 2’-deoxynucleoside and triphosphate. The role of OsSAMHD1 in nucleotide catabolism has been inferred based on sequence homology and nucleotide supplementation experiments (Wang et al., 2022); however, its enzymatic function remains uncharacterised. *In vitro* analysis has revealed that His-tagged VEN4 can convert dGTP to 2-dG (Lu et al., 2023). A recent study has shown that *Arabidopsis* VEN4 is involved in DNA double-strand break (DSB) repair through homologous recombination (HR), in which the deacetylation of a conserved lysine residue, K354 in human SAMHD1 and K248 in VEN4, plays a critical role (Sarmiento-Mañús et al., 2025). This conserved lysine residue is also present in OsSAMHD1, suggesting it may participate in this repair process. However, the role of OsSAMHD1 in DSB repair has not yet been characterised. Nonetheless, a proper balance of nucleotides is required for cells to function effectively. An imbalance of nucleotides can impair DNA and RNA synthesis, repair mechanisms, and genome stability, as nucleotides are fundamental elements of DNA and RNA and essential components of metabolic cofactors.

Moreover, the substitution at R310 in OsSAMHD1 is equivalent to R216 in *Arabidopsis* VEN4 and R318 in human SAMHD1 (**Supplementary Figure 6**). Notably, Human SAMHD1 is active in the tetramer state, containing allosteric sites I and II, as well as catalytic sites (Ji et al., 2014). The mutated position is close to important residues D311, D330, and R333 in human SAMHD1. D311 is one of the conserved histidine-aspartate residues and plays a critical role in metal ion coordination at the enzyme’s catalytic site (Ji et al., 2014). D330 is a vital residue located at allosteric site II. R333 is critical in stabilising dATP binding at the allosteric site II by forming a salt bridge with E355 (Ji et al., 2014). Therefore, we speculate that R310H mutation may disrupt these conserved interactions and affect structural integrity, eventually leading to an imbalanced dNTP pool and defective chloroplast development.

### 4.3 Interaction between OsRNRS1 and OsSAMHD1

RNA-seq analysis revealed the upregulation of *OsRNRS1* in RBR05 compared to solid green leaf Riceberry. RNR is the rate-limiting enzyme in the *de novo* synthesis of dNDPs, catalysing the reduction of ribonucleotide diphosphates (rNDPs) to deoxyribonucleotide diphosphates (dNDPs) in the cytosol. These dNDPs are subsequently converted into deoxyribonucleotide triphosphates (dNTPs), the building blocks of DNA. Rice RNR comprises two large and two small subunits, forming α2β2 heterodimers (Yoo et al., 2009). *RNRS1* encodes the small subunit of ribonucleotide reductase (RNR). The mutation of *OsRNRS1* results in abnormal chloroplast development, white-striped leaf, altered cell cycle progression, drooping leaf, narrow leaf, dwarfism, and deformed floral organs, depending on the degree and position of mutation (Yoo et al., 2009; Chen et al., 2015; Qin et al., 2017; Shen et al., 2023). We have shown that the upregulation of *OsRNRS1* was associated with a white-striped leaf phenotype in RBR05.

Interestingly, the phenotype of RBR05, characterised by white-striped leaves and aberrant chloroplast development, is similar to that of both *OsSAMHD1* and *OsRNRS1* mutants. These mutants, including RBR05, exhibit temperature-sensitive phenotypes, highlighting the importance of *OsRNRS1* and *OsSAMHD1* in chloroplast development under low-temperature (restrictive) conditions (Yoo et al., 2009; Wang et al., 2022). The upregulation of *OsRNRS1* and *OsSAMHD1* at low temperatures suggest a requirement for their function in such conditions. In the *st3* mutant, chloroplast development-related genes were downregulated compared to the wild type under low temperature, indicating a disruption in chloroplast biogenesis at the molecular level (Wang et al., 2022). Additionally, the yeast complementation assay of *OsRNRL1* in yeast *rnr1rnr3* knockout line has revealed that OsRNRL1 activity is thermosensitive (Yoo et al., 2009). Notably, the mutation in *OsRNRL1*, which encodes the large subunit of ribonucleotide reductase, also leads to white-striped leaf phenotype and temperature sensitivity. Similarly, OsSAMHD1 activity may also be thermosensitive—reduced or unstable under low-temperature conditions—as observed for OsRNRL1. The R310H mutation in OsSAMHD1 may compromise its structural integrity. Taken together, R310H mutation may prevent OsSAMHD1 from meeting the increased demand to balance the nucleotide pool under low temperature conditions, leading to more pronounced defects in chloroplast development. However, precise biochemical mechanisms of OsSAMHD1 and its R310H mutant under restrictive temperature remain to be elucidated. Future investigation of OsSAMHD1 thermostability will provide insight into how low temperatures affect its function, thereby linking biochemical behaviour to the observed phenotype.

A functional connection between these two enzymes, SAMHD1 and RNR, has been reported (Ji et al., 2014; Sarmiento-Mañús et al., 2023). Ji et al. (2014) elucidated the structural basis for SAMHD1 regulation, revealing the similarity in enzymatic properties between SAMHD1 and RNR. Both enzymes function as oligomers and contain allosteric sites I and II and catalytic sites, enabling them to sense and respond to changes in cellular nucleotide concentrations. RNR is activated by ATP binding at the allosteric site 1, but is inhibited by dATP. In contrast, dATP serves as a key activator for SAMHD1. It has the highest affinity among dNTPs for allosteric site 2; notably, SAMHD1’s allosteric site 1 exhibits specificity for GTP or dGTP (Ji et al., 2014). A recent study has provided additional evidence supporting the role of SAMHD1 in dNTP degradation and the maintenance of dNTP homeostasis, as well as its functional interconnection with nucleotide biosynthesis (McCown et al., 2025). The catalytic efficiency of SAMHD1 is modulated by the identity of dNTP occupying its allosteric sites. For example, the availability of purine dNTPs can promote the depletion efficiency of pyrimidine dNTPs. Therefore, the elevated levels of specific dNTPs—resulting from increased *de novo* biosynthesis (by RNR) or the salvage pathway—can enhance the degradation of other dNTP species (McCown et al., 2025).

A study in *Arabidopsis* has further demonstrated a genetic interaction between *VEN4* and *TSO2* (Sarmiento-Mañús et al., 2023), which are homologs of rice *OsSAMHD1* and *OsRNRS1*, respectively. *TSO2* appeared to play a more critical role in dNTP metabolism than *VEN4*, as suggested by differences in phenotype observed in reciprocal sesquimutants (Sarmiento-Mañús et al., 2023). In addition, coordination between *OsSAMHD1* and genes in both the *de novo* and salvage nucleotide biosynthetic pathways has been discussed in the *st3* mutant, which carries a mutation in *OsSAMHD1* (Wang et al., 2022). In the *st3* mutant, the decreased expression of *OsSAMHD1* and *de novo* biosynthetic genes, including *OsRNRS1*, was observed in parallel with the increased expression of salvage pathway genes (Wang et al., 2022), suggesting a compensatory adjustment in nucleotide metabolism. Our findings, along with the studies in *Arabidopsis* and *st3* mutant, emphasise the functional interdependence of *OsSAMHD1* and *OsRNRS1* in nucleotide metabolism, genome stability and maintaining chloroplast integrity. Although nucleotide content was not directly quantified in our study, the decrease in dGTP has been reported in the *st3* mutant (Wang et al., 2022), supporting the idea of disrupted nucleotide homeostasis. Incorporating nucleotide profiling in future investigation would help clarify the biochemical relationship between these genes and their role in maintaining nucleotide homeostasis.

Notably, we did not find a mutation in *OsRNRS1* based on the QTL-seq analysis, nor did we observe differential gene expression of *OsSAMHD1* between RBR05 and RB in the RNA-seq analysis. The expression patterns of *OsSAMHD1* and *OsRNRS1* have been studied in two *OsSAMHD1* mutants, *st3* and *wsl214* (Wang et al., 2022; 2023). While both mutants exhibited decreased expression of *OsSAMHD1*, *OsRNRS1* expression was increased in *wsl214* but decreased in *st3*. The variation in expression patterns may reflect differences in the severity of mutations and their impact on OsSAMHD1 protein function. Notably, *wsl214* carries a missense mutation, similar to RBR05, whereas *st3* carries a mutation that leads to an alternative transcript variant. In *Arabidopsis*, the transcript abundance of *VEN4*, a homolog of *OsSAMHD1*, remained unchanged in the *ven4-0* mutant, which carries a point mutation E249L (Sarmiento-Mañús et al., 2023). The E249L substitution occurs at a critical residue required for salt-bridge formation, altering the structural rigidity of the VEN4 protein. This finding demonstrates that the mutation can disrupt protein function without altering transcript abundance, highlighting the importance of post-transcriptional and post-translational regulation. In the case of SAMHD1, enzymatic activity is tightly controlled by allosteric regulation through nucleotide binding as discussed above. In addition, post-translational modifications have been reported in humans SAMHD1 (Coggins et al., 2020).

The increased expression of *OsRNRS1* in our study was probably a compensatory mechanism in response to the disruption of cytosolic nucleotide metabolism caused by malfunctional OsSAMHD1. A similar interplay among genes involved in nucleotide metabolism has been described in the *st3* (Wang et al., 2022). The functional interaction between *OsSAMHD1* and *OsRNRS1* may imply the essence of a balanced nucleotide pool for proper cellular function. Along with *OsRNRS1*, we also identified upregulation of *OsDRP5B*, *Dynamin-Related Protein 5B*, also known as *Accumulation and Replication of Chloroplasts 5 (ARC5)*, which encodes a dynamin-related protein GTPase, a cytosolic component of the chloroplast division machinery. Therefore, maintaining the nucleotide pools is essential for chloroplast division and propagation during leaf development.

### 4.4 Abnormal chloroplast development and the repression of chlorophyll biosynthesis and photosynthetic apparatus formation

The white-striped leaf phenotype in the RBR05 was primarily caused by aberrant chloroplast development. This defect led to the formation of non-chlorophyllous mesophyll cells characterised by their transparent appearance and lack of chlorophyll. These cells were found randomly throughout the leaf tissue. Consistent with the aberrant chloroplast ultrastructure, transcriptome analysis revealed the downregulation of *OsLhcb1.1*, the *AtCURT1B* homolog, and *cytochrome b6-f complex subunit 4* in RBR05, suggesting the suppression of photosynthetic apparatus formation. AtPsaP/AtCURT1B can form oligomers with AtCURT1A, AtCURT1C, and AtCURT1D to modulate the grana structure by inducing membrane curvature at the grana margin (Armbruster et al., 2013).

Chlorophyll is the primary photosynthetic pigment and a significant factor in determining leaf greenness. In this study, *OsPORA*, which encodes one of the two isoforms of protochlorophyllide reductase (POR), was the only gene in the chlorophyll biosynthetic pathway that exhibited differential expression between the solid green leaf Riceberry and RBR05 (downregulated in RBR05). POR catalyses light-dependent reduction of protochlorophyllide, a photosensitive compound, into chlorophyllide. Their expression analysis has suggested that *OsPORA* primarily functions during the early stage of leaf development, whereas *OsPORB* maintains a threshold level of chlorophyll throughout leaf development (Sakuraba et al., 2013; Kwon et al., 2017). Although the function of *OsPORA* is not fully understood, the decreased expression of *OsPORA* points to a reduction in chlorophyll biosynthesis, which contributes to the reduced chlorophyll content and the presence of non-chlorophyllous cells in RBR05. Chlorophyll metabolism and the assembly of photosynthetic apparatus are tightly synchronised. Chlorophylls form pigment-protein complexes, which are embedded in the thylakoid membranes. Unbound chlorophyll and its intermediates can induce photooxidative damage (Sun et al., 2011; Wang et al., 2015; Liu et al., 2022, 2023; Zhao et al., 2023). Several studies have shown that the disruption of chlorophyll biosynthetic genes cause not only a decline in photosynthetic pigment content but also aberrations in chloroplast ultrastructure (Zhang et al., 2006, 2015; Liu et al., 2007, 2021a; Wang et al., 2010, 2017b, 2021; Tian et al., 2013; Ruan et al., 2017; Long et al., 2022; Shim et al., 2023; Yao et al., 2023).

Interestingly, an interconnection between POR and CURT1 has been demonstrated. Both are the components of the prolamellar body (PLB) in the etioplast and play vital roles in chloroplast development during de-etiolation (Sandoval-Ibáñez et al., 2021; Liang et al., 2022). Notably, POR proteins are the most abundant protein in etioplast (Blomqvist et al., 2008; Floris and Kühlbrandt, 2021). Disruption of PLB organisation was reported in *Arabidopsis PORA* and *PORB* mutants (Franck et al., 2000; Frick et al., 2003; Paddock et al., 2012). In addition to regulating thylakoid margin architecture, a recent study revealed that CURT1 also contributes to maintaining PLB structure and modulating the maturation of thylakoid membrane during de-etiolation (Sandoval-Ibáñez et al., 2021; Liang et al., 2022). The *CURT1* mutant exhibited PLB disorganisation and decreased POR accumulation (Sandoval-Ibáñez et al., 2021).

### 4.5 Increased chloroplast gene expression and chloroplast division

RBR05 showed increased expression of genes related to plastid gene transcription (*OsFLN2*), post-transcriptional processing (*Chloroplastic group IIA intron* sp*licing facilitator CRS1* and *OsTRZ2*), and translation (*Chloroplast 50S ribosomal protein L16*). Functional studies and phenotypic characterisation of these genes or their homologs have demonstrated their critical roles in chloroplast development and leaf colouration. For example, *OsFLN2* mutants exhibited albino or white-striped phenotypes (Qiu et al., 2018). *OsTRZ2* knockout mutant displayed a seedling-lethal albino phenotype, while its knockdown mutant showed pale green leaves (Long et al., 2013). The increased expression of these genes, along with the chloroplast division gene *OsDRP5B/OsARC5*, suggests that RBR05 may enhance the expression of chloroplast-encoded genes and chloroplast division as compensatory mechanisms to alleviate the defective chloroplast development in the white leaf region. Meanwhile, OsFLN2 was reported to interact with thioredoxin OsTrx-Z, a PEP-associated protein, forming a TRx-FLN regulatory complex to regulate PEP-dependent gene expression (Qiu et al., 2018). Additionally, a recent study has revealed the interaction between OsSAMHD1 and catalase OsCATC, as well as an increased accumulation of reactive oxygen species (ROS) in the OsSAMHD1 mutant, pointing to the critical role of OsSAMHD1 in maintaining cellular ROS homeostasis (Wang et al., 2023). Taken together, the upregulation of *OsFLN2* and other genes involved in chloroplast gene expression is probably not only compensating for the malfunctioning chloroplast development but also fine-tuning the expression of PEP-dependent genes in response to the altered chloroplast redox status in RBR05.

## 5 Conclusions

**Figure 7:**
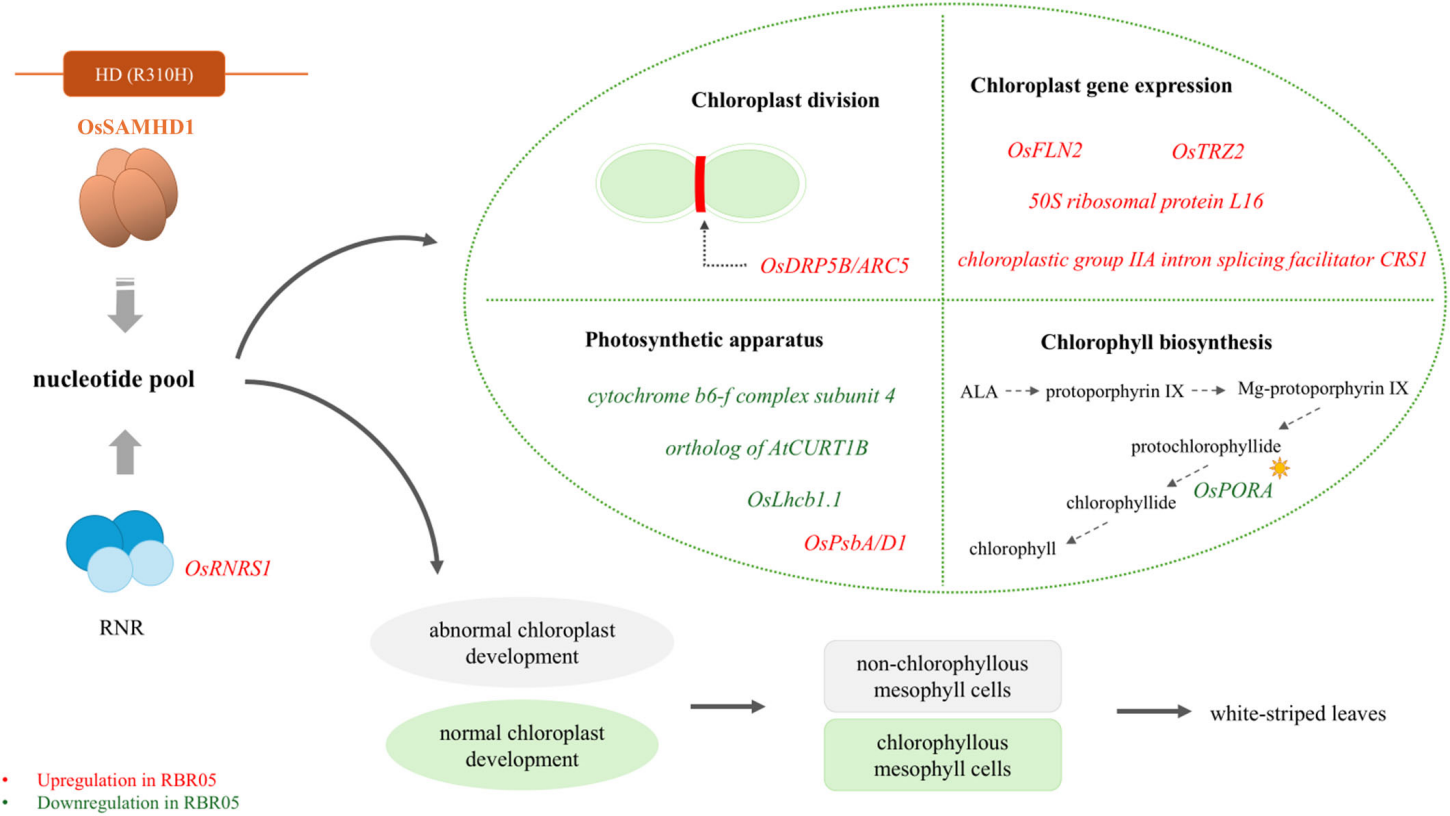
Proposed working model of the *OsSAMHD1* R310H mutation and its impact on white-striped leaf phenotype in RBR05. RBR05 carries an R310H substitution in OsSAMHD1, which is proposed to function in nucleotide catabolism. This mutation may disrupt nucleotide pool homeostasis, resulting in defective chloroplast development in non-chlorophyllous mesophyll cells and leading to the white-striped leaf phenotype. This phenotype is associated with the downregulation of *OsPORA*, a chlorophyll biosynthetic gene, and genes encoding components of the photosynthetic apparatus. The upregulation of genes associated with chloroplast gene expression and chloroplast division likely reflects a response to counteract impaired chloroplast development. In addition, the upregulation of *OsRNRS1*, which encodes the small subunit of ribonucleotide reductase (RNR), a rate-limiting enzyme in the de novo biosynthesis of nucleotides, may represent a compensatory mechanism in response to nucleotide pool imbalance.

This study has identified a novel mutation (R310H) in OsSAMHD1 as a key modulator of the white-striped phenotype in RBR05. The mutant exhibited transient expression of a white-striped leaf; that is, seedlings normally have green leaves and become white-striped during the tillering stage, and the white-striped area expands when the temperature is below 25°C. The mutation resulted in abnormal chloroplast development, a lack of chlorophyll pigment, and the formation of non-chlorophyllous cells in the white region of the leaves. Transcriptome analysis revealed that this phenotype was associated with the decreased expression of genes involved in the formation of photosynthetic machinery and chlorophyll biosynthetic pathway. The increased expression of genes related to chloroplast-encoded gene expression and chloroplast division were probably a compensatory mechanism to cope with the aberrant chloroplast development in the white leaf sector. The upregulation of *OsRNRS1* and the mutation observed in *OsSAMHD1* highlight the critical role of maintaining nucleotide homeostasis and proper chloroplast development. We proposed a working model describing the essential roles of *OsRNRS1* and *OsSAMHD1* in controlling the novel white-striped leaf phenotype in RBR05 rice (**Figure 7**). However, further research is needed to validate the function of *OsSAMHD1* and its interconnection with *OsRNRS1*.

## Data Availability

The datasets supporting the conclusions of this article are included within the article and its supplementary files. The sequencing data are available at NCBI (https://www.ncbi.nlm.nih.gov/) with accession number PRJNA1279394.
