# Supplementary figures and images for: Insight into the genetics of a novel white-striped leaf in rice
Source: Front Plant Sci. 2025 Aug 20;16:1622640. doi: 10.3389/fpls.2025.1622640 (PMC12405276; doi:10.3389/fpls.2025.1622640)

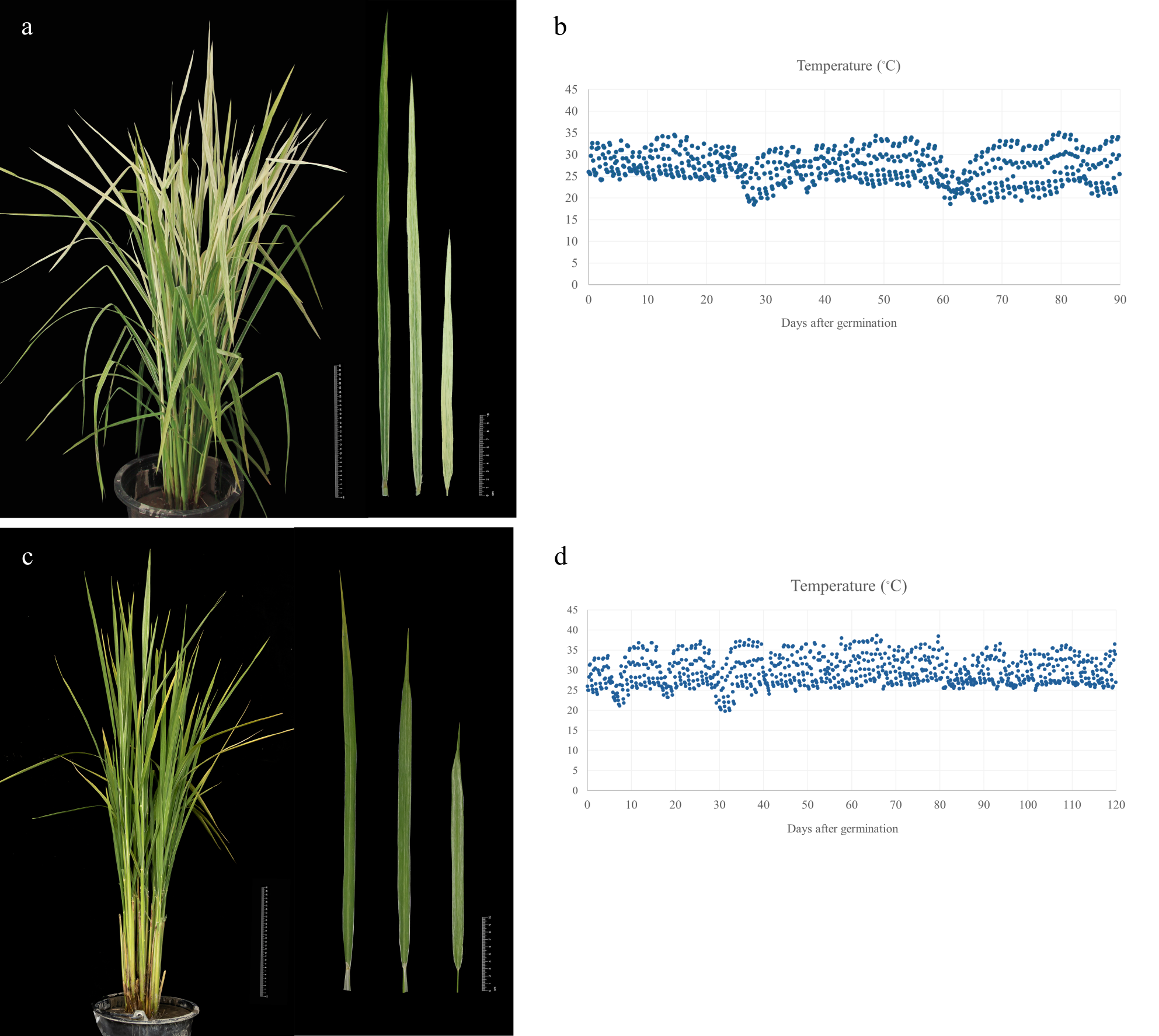

Supplement: Supplementary file 1 [file Image1.tif]

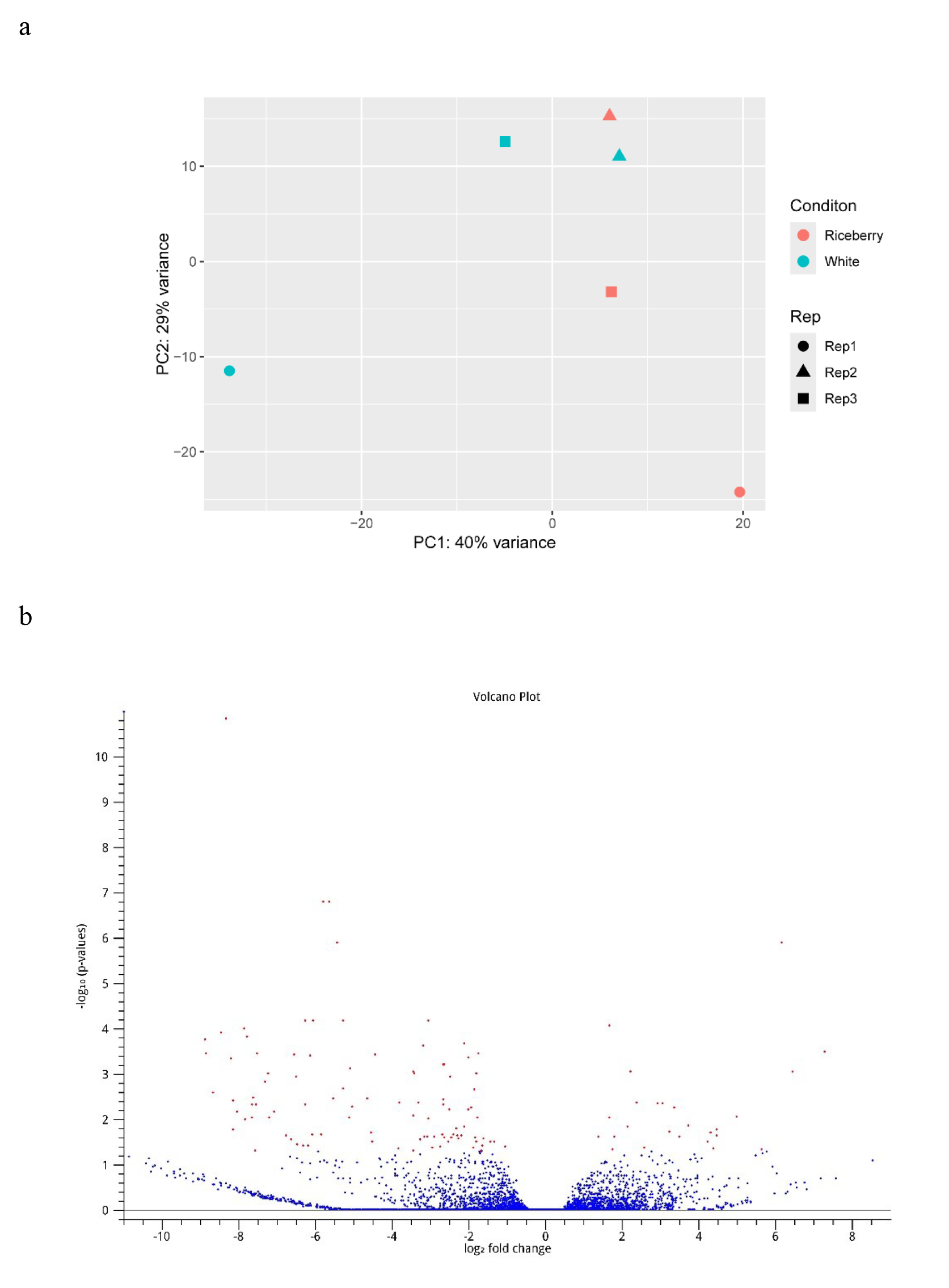

Supplement: Supplementary file 2 [file Image2.tif]

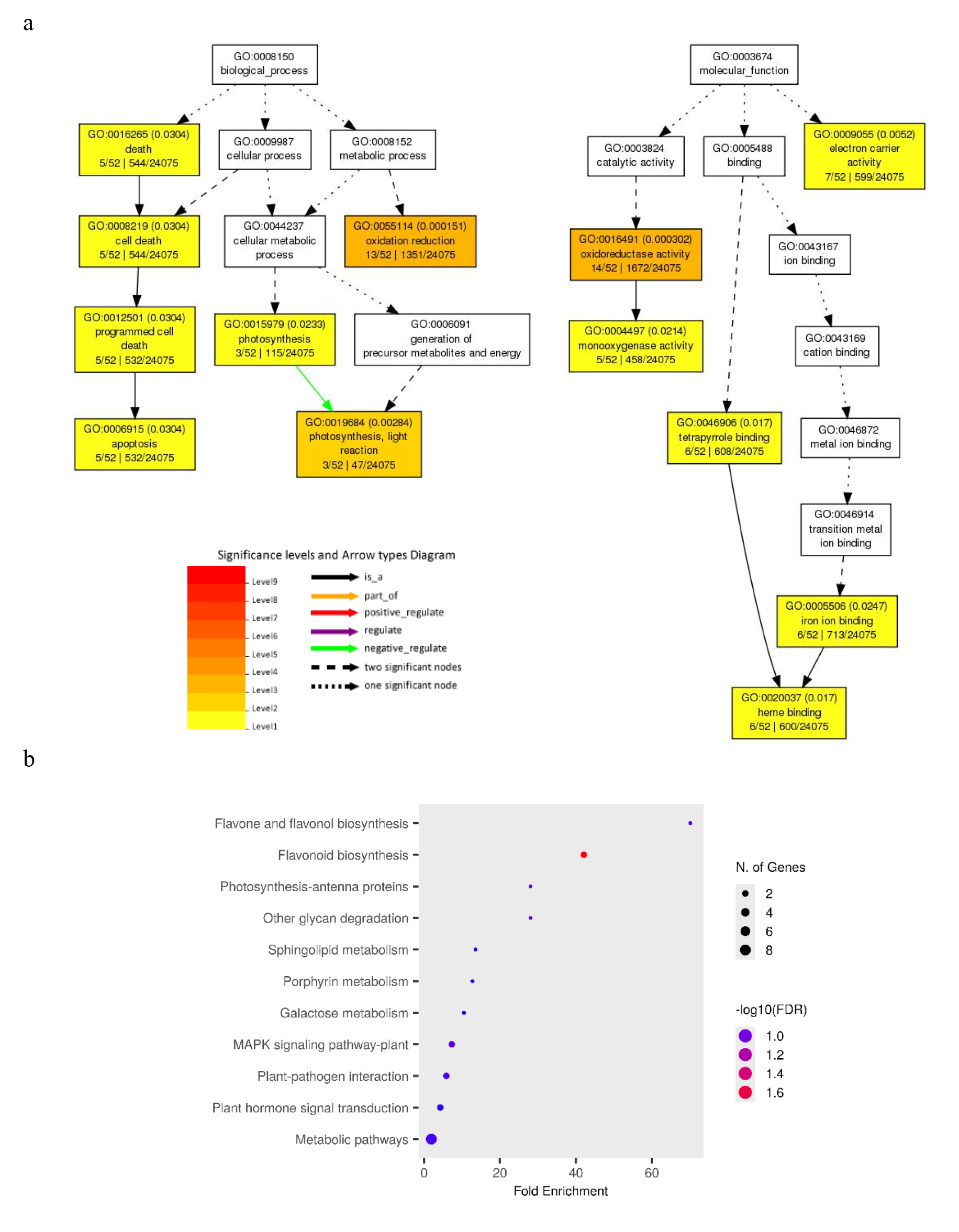

Supplement: Supplementary file 3 [file Image3.tif]

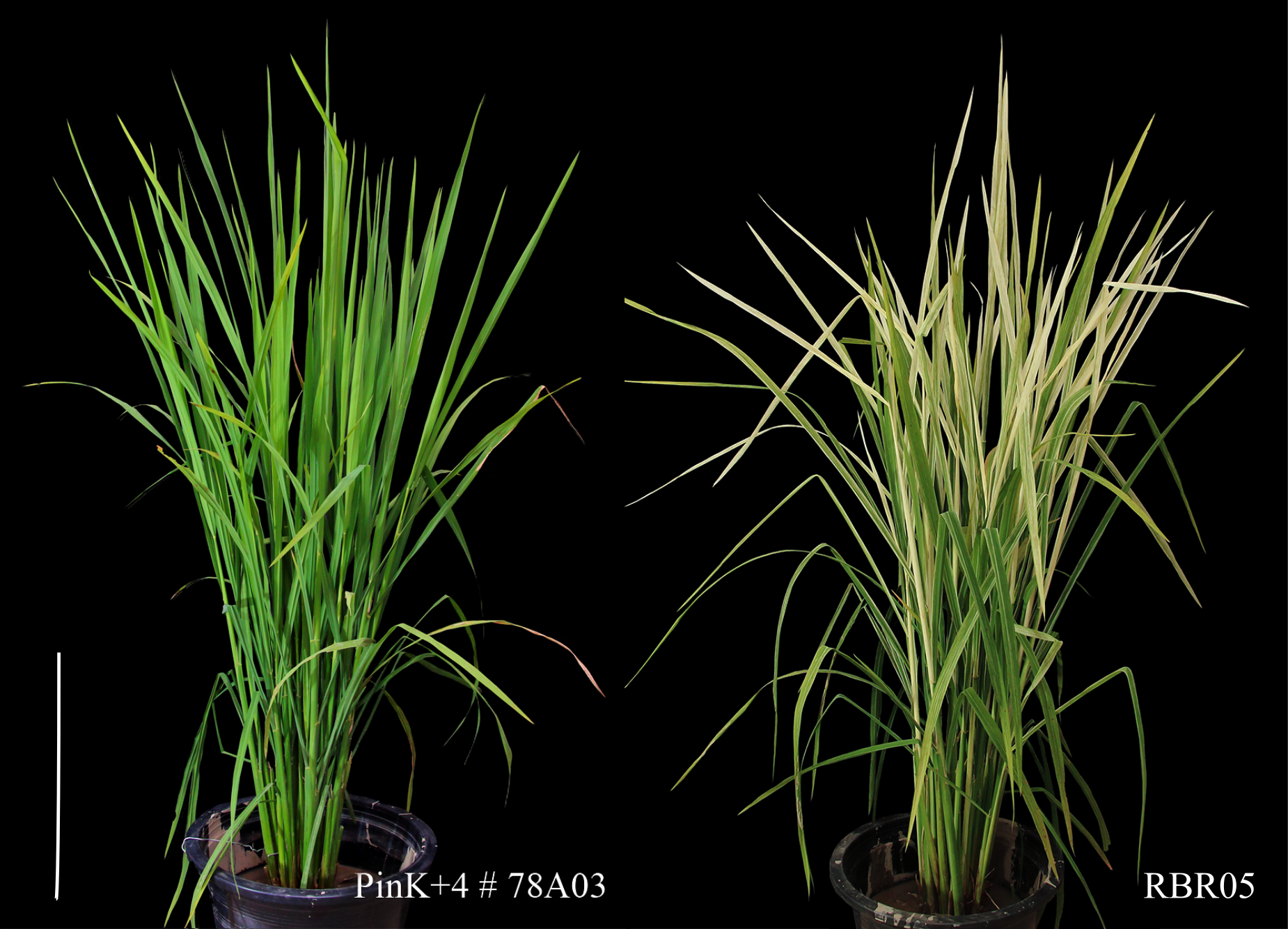

Supplement: Supplementary file 4 [file Image4.tif]

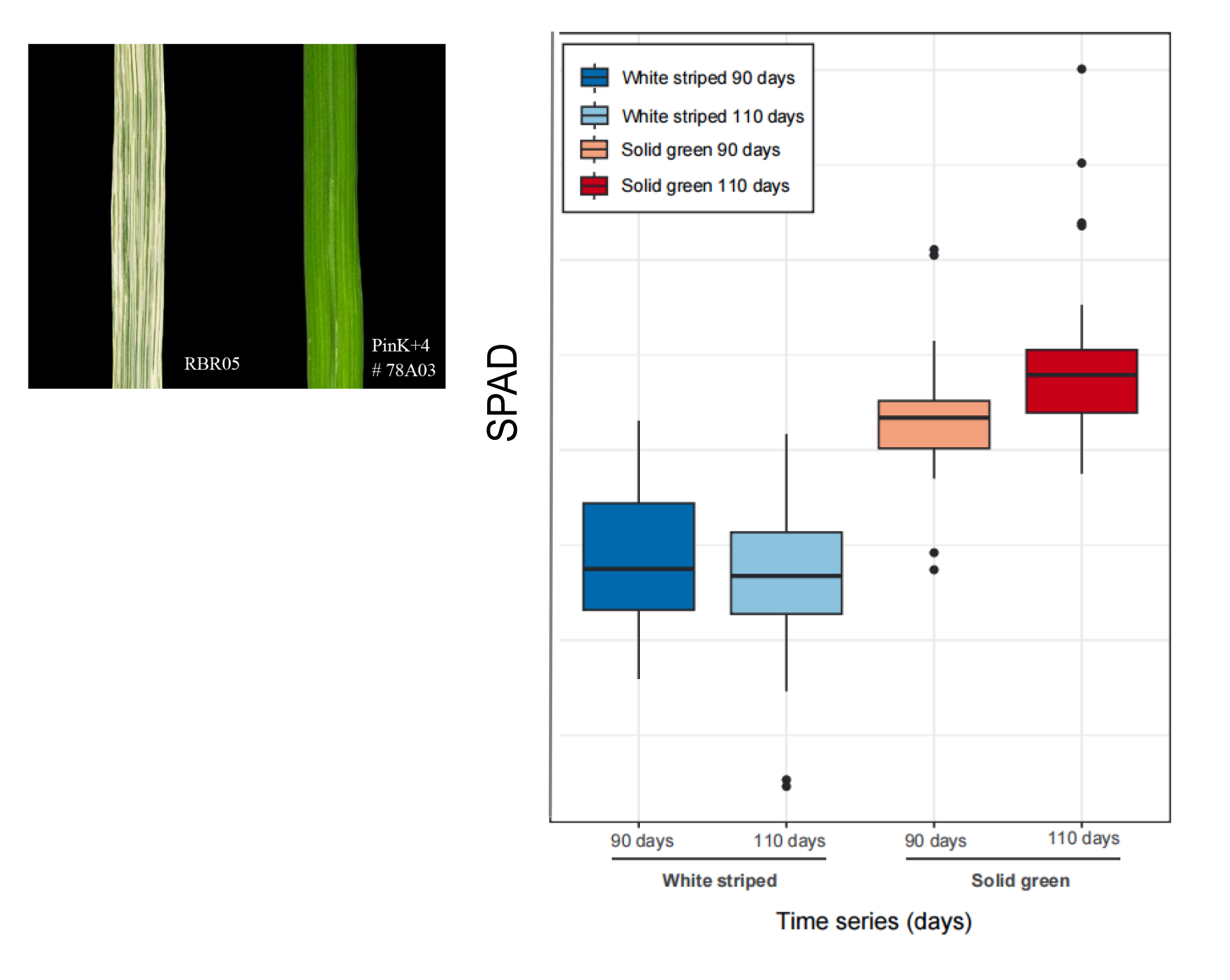

Supplement: Supplementary file 5 [file Image5.tif]

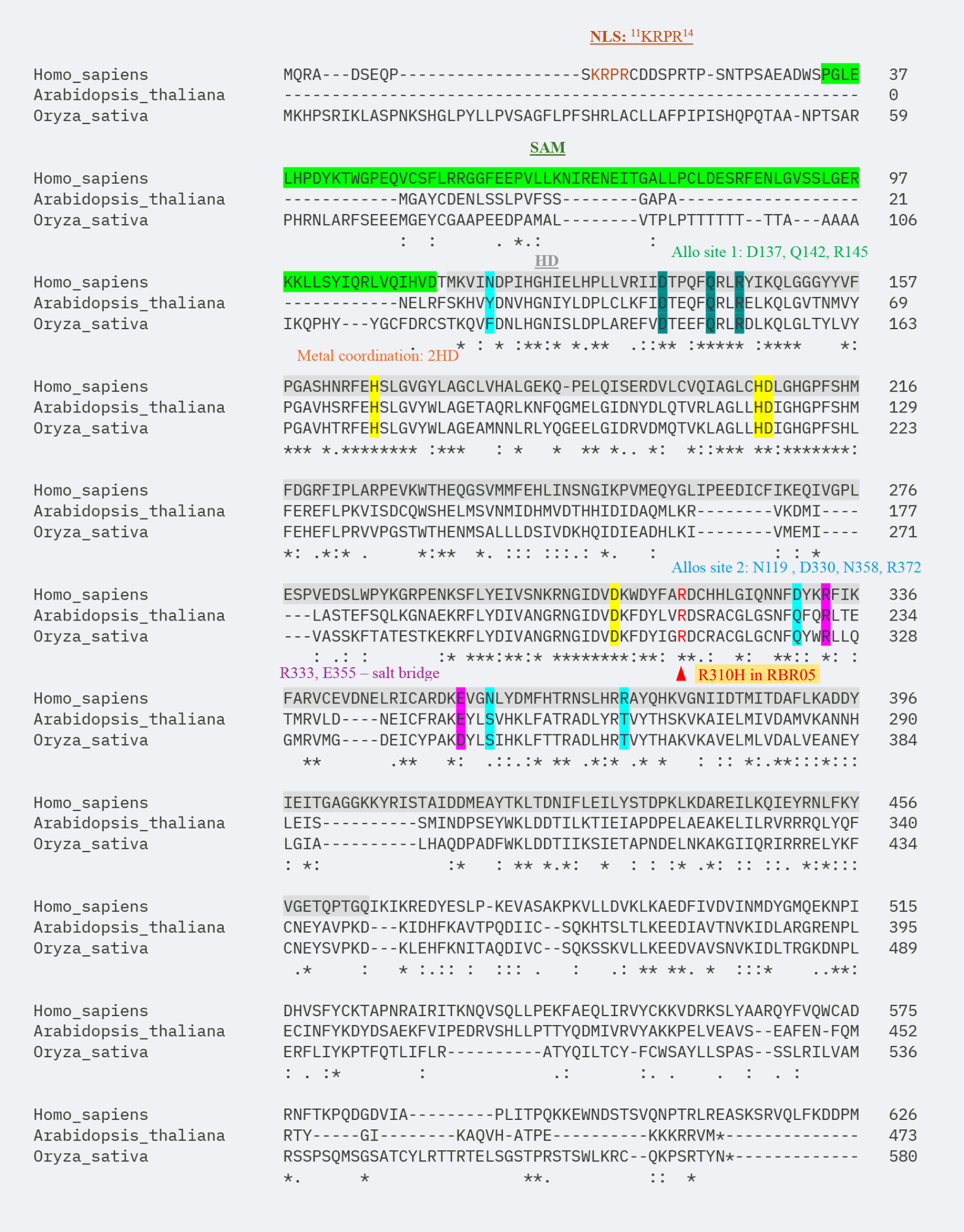

Supplement: Supplementary file 6 [file Image6.tif]
