# Supplementary material for: Insight into the genetics of a novel white-striped leaf in rice
Source: Front Plant Sci. 2025 Aug 20;16:1622640. doi: 10.3389/fpls.2025.1622640 (PMC12405276; doi:10.3389/fpls.2025.1622640)
Supplement: Supplementary file 8 [file Table2.docx]

**Supplementary data**

**Supplementary Figure 1** Temperature-sensitive phenotype of white-striped leaf Rainbow rice 05 (RBR05)

**Supplementary Figure 2** Principal component analysis (PCA) of RNA-seq data and volcano plot of differentially expressed genes (DEGs)

**Supplementary Figure 3** GO and KEGG enrichment of differentially expressed genes (DEGs)

**Supplementary Figure 4** The parental lines, PinK+4 # 78A03 and RBR05, used for genetic analysis of white-striped trait and QTL-seq.

**Supplementary Figure 5** Leaf phenotype and SPAD chlorophyll readings in RBR05, PinK+4 #78A03, SGL bulk, and *wsl* bulk.

**Supplementary Figure 6** Multiple sequence alignment of SAMHD1 protein across human (*Homo sapiens*), *Arabidopsis thaliana*, and rice (*Oryza sativa*)

**Supplementary Table 1** List of differentially expressed genes in RBR05 compared to Riceberry (excel file)

**Supplementary Table 2** List of significantly enriched GO terms and their associated genes (excel file)

**Supplementary Table 3** List of KEGG pathways and their statistical values (excel file)

**Supplementary Table 4** Evaluation of leaf colour characteristics of white-striped and solid green rice groups by visual assessment and using the chlorophyll meter SPAD to examine the chlorophyll content

**Supplementary Table 5** Genetic analysis of the *qwsl1ch1_503564* mutant

**Supplementary Table 6** Summary of whole-genome sequencing data of parental lines, solid green leaf (SGL) and white-striped leaf (*wsl*) bulks

**Supplementary Table 7** Chromosome-wise distribution of common single nucleotide polymorphisms (SNPs) and Insertions-Deletion (Indels)

**Supplementary Table 8** Candidate genes with delta SNP index ≥ 0.5 and moderate or high impact (excel file)


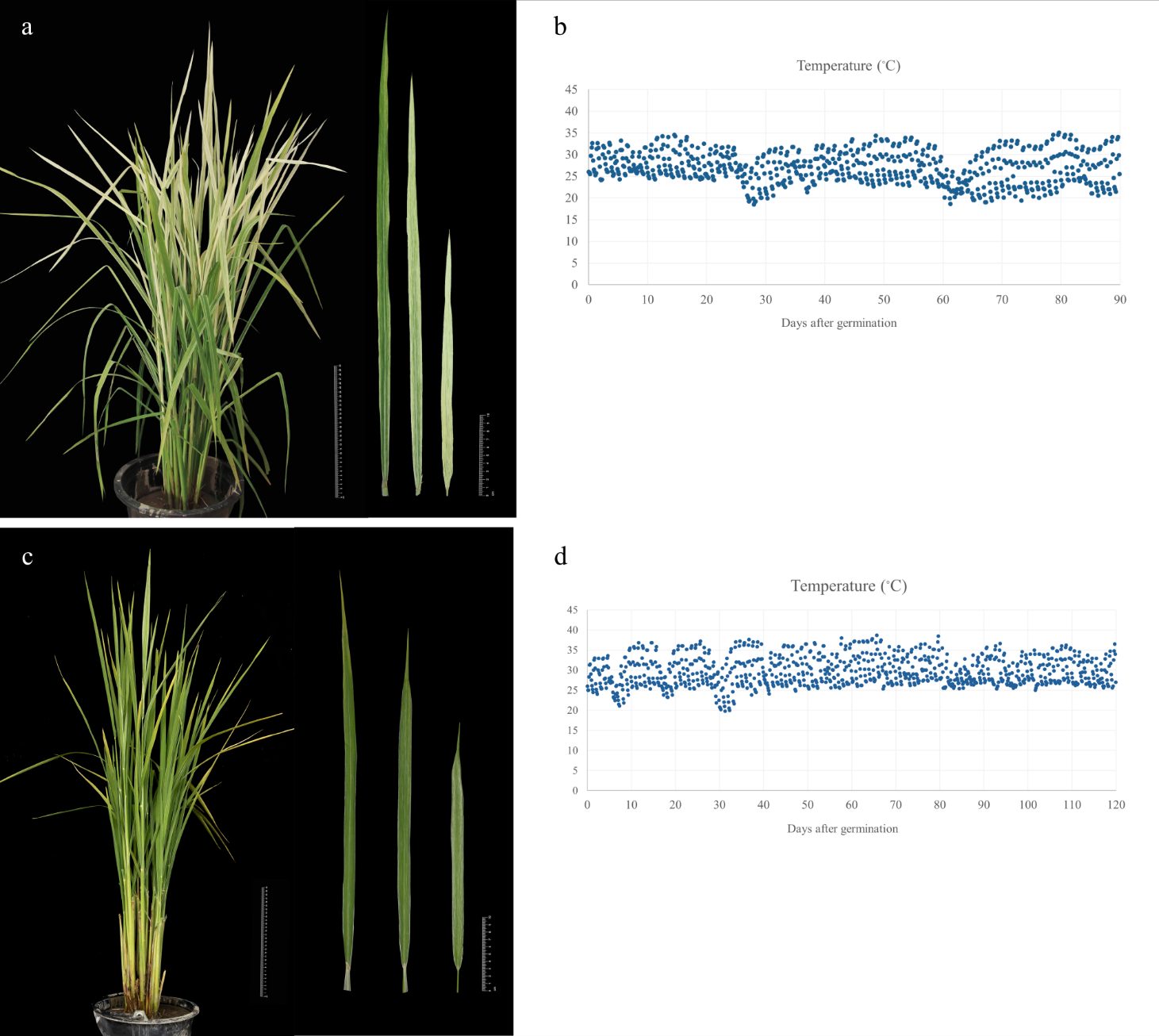


**Supplementary Figure 1:** Temperature-sensitive phenotype of white-striped leaf Rainbow rice 05 (RBR05). **(a, c)** Phenotypes of RBR05 plants grown under natural paddy field conditions during two different seasons: **(a)** winter (late October to January) and **(c)** summer (late February to June). Each panel shows a whole plant (left) and the three uppermost leaves (right) at the early booting stage, approximately 90 days after germination (DAG) in winter and 120 DAG in summer. From right to left: flag leaf (F), first (-1), and second (-2) leaves below the flag leaf. Notably, RBR05 exhibited delayed flowering in the summer season compared to winter. **(b, d)** Daily ambient temperatures recorded during the growth periods of plants shown in **(a)** and **(c),** respectively. Scale bar = 30 cm (whole plants); 10 cm (leaves).


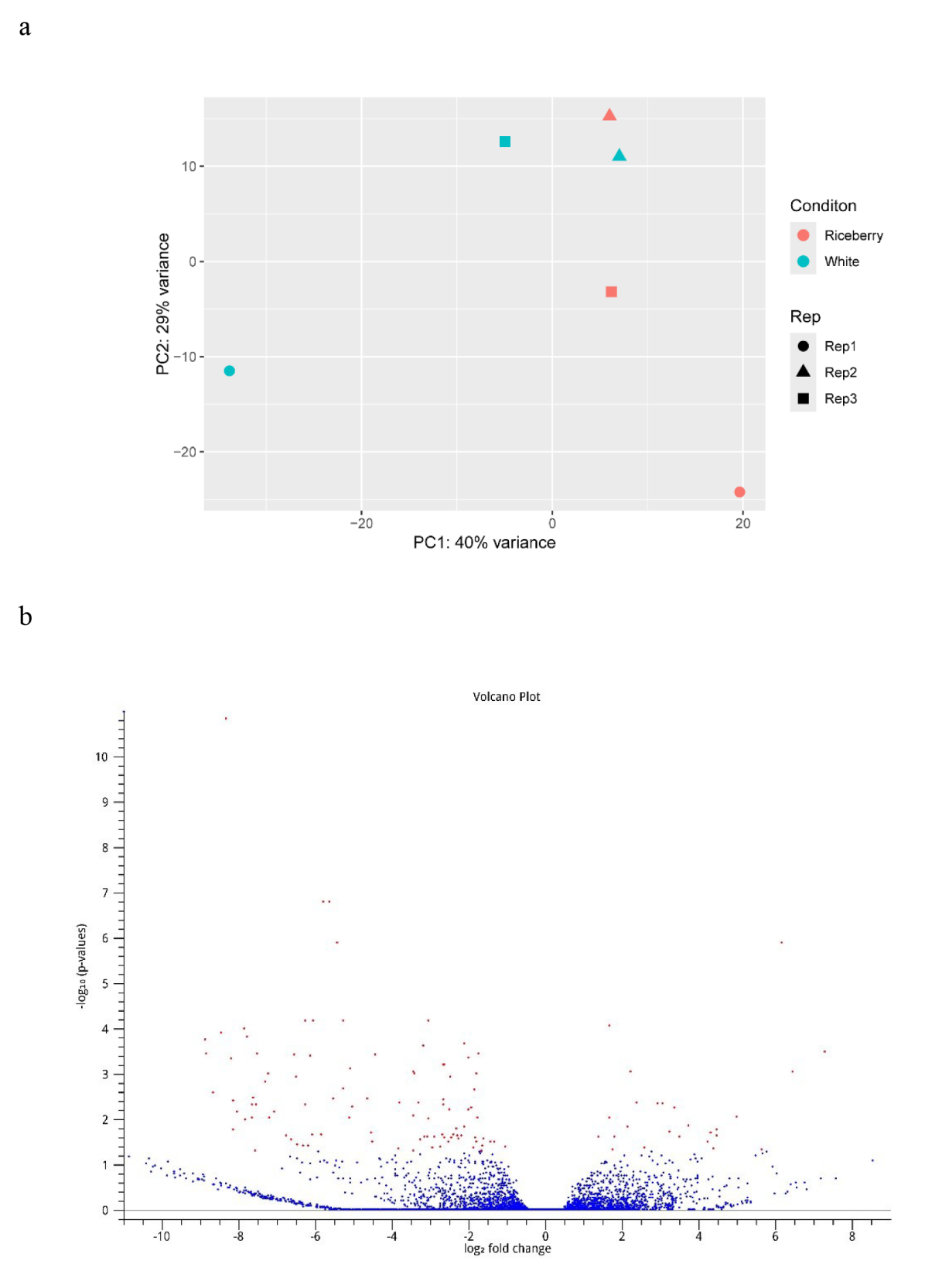


**Supplementary Figure 2:** Principal component analysis (PCA) of RNA-seq data and volcano plot of differentially expressed genes (DEGs). **(a)** PCA showing sample clustering; notably, “white” refers to RBR05. **(b)** Volcano plot of DEGs; red dots represent genes that are significantly differential expressed between Riceberry and RBR05.


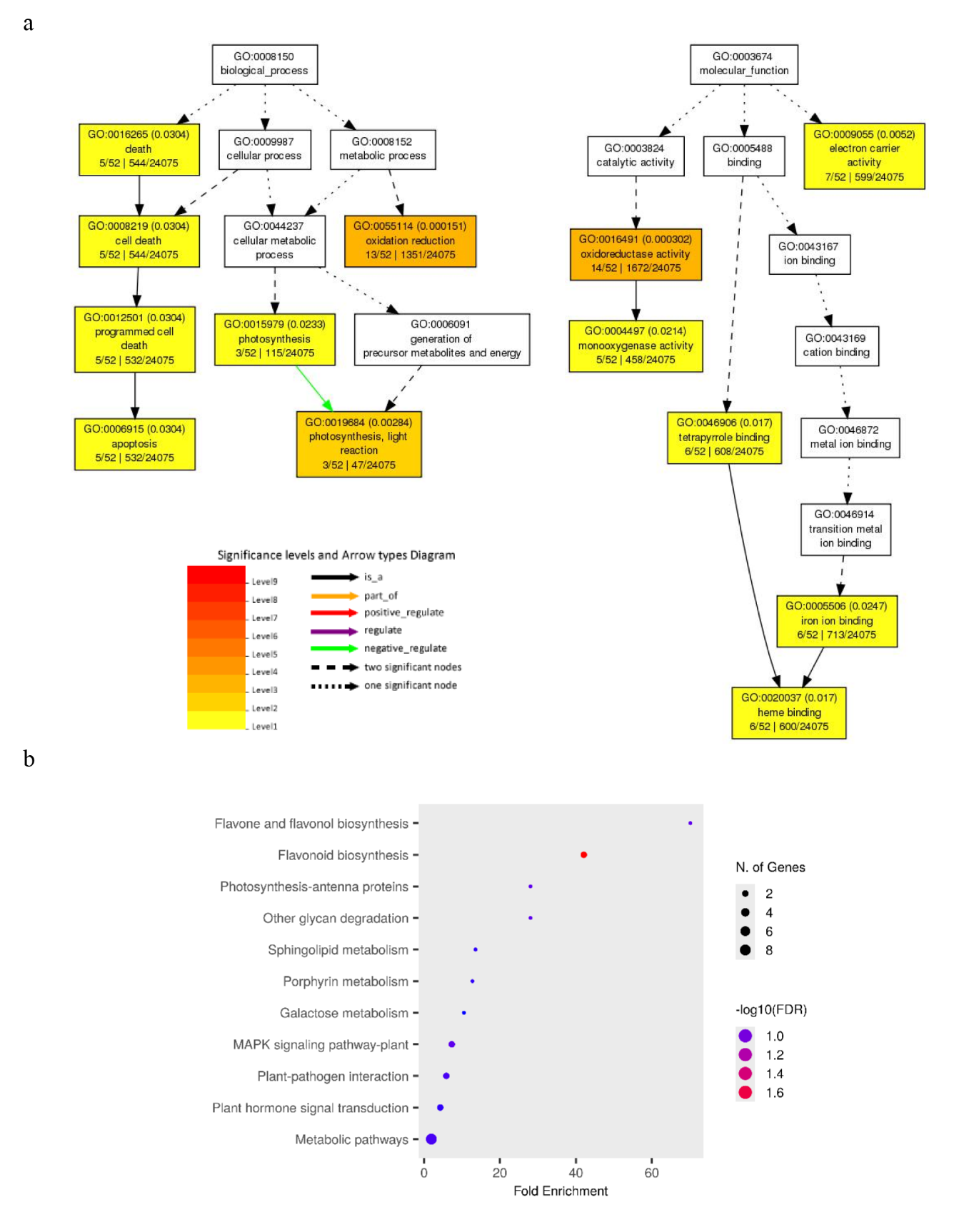


**Supplementary Figure 3:** GO and KEGG enrichment of differentially expressed genes (DEGs). **(a)** GO terms for biological process (left) and molecular function (right); significantly enriched GO terms are highlighted. (**b)** KEGG pathway enrichment showing several pathways along with their -log_10_(FDR) value; notably, only flavonoid biosynthesis was significantly enriched (FDR < 0.05).


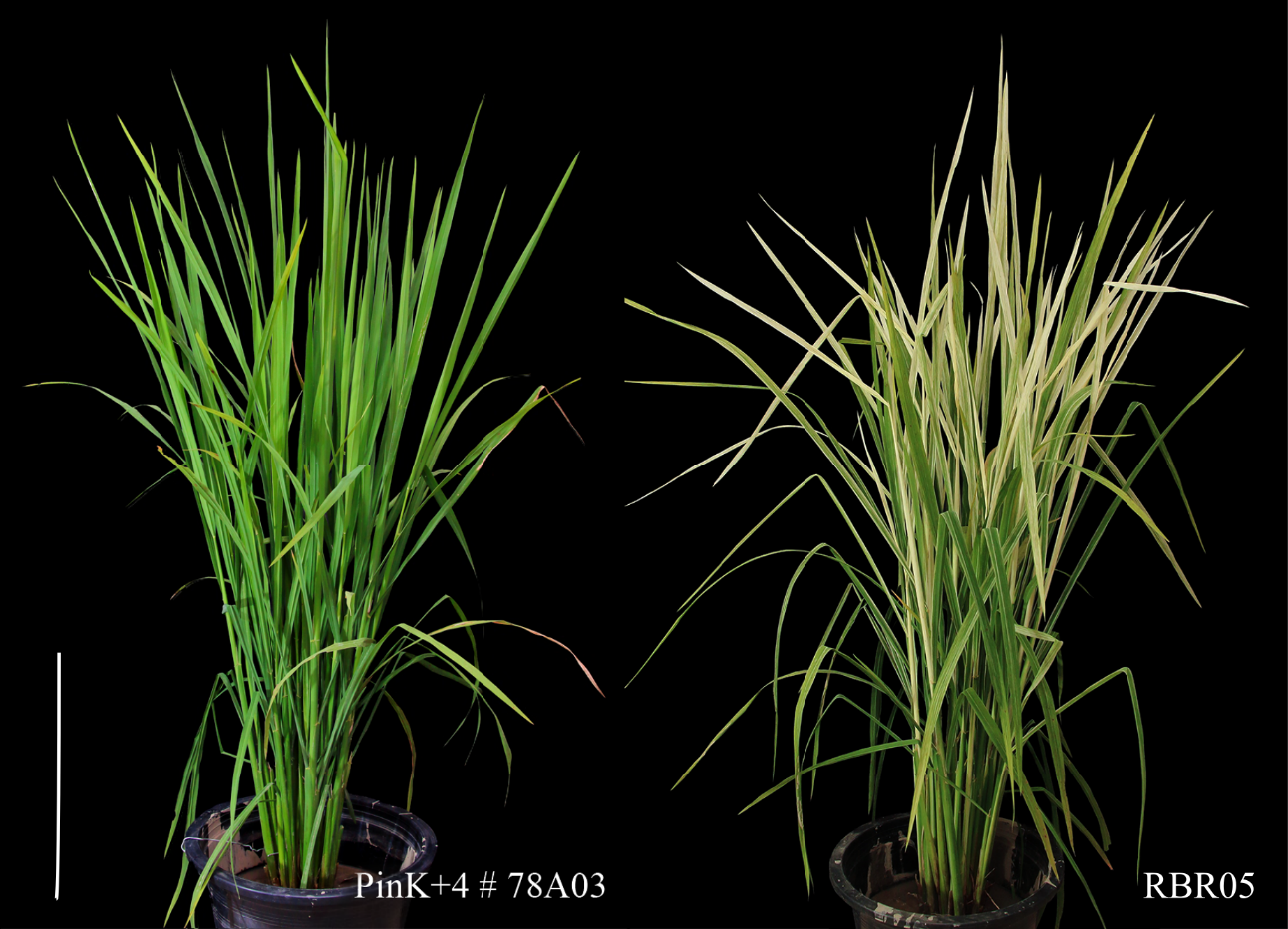


**Supplementary Figure 4:** The parental lines, PinK+4 # 78A03 and RBR05, used for genetic analysis of white-striped trait and QTL-seq. Scale bar = 30 cm.


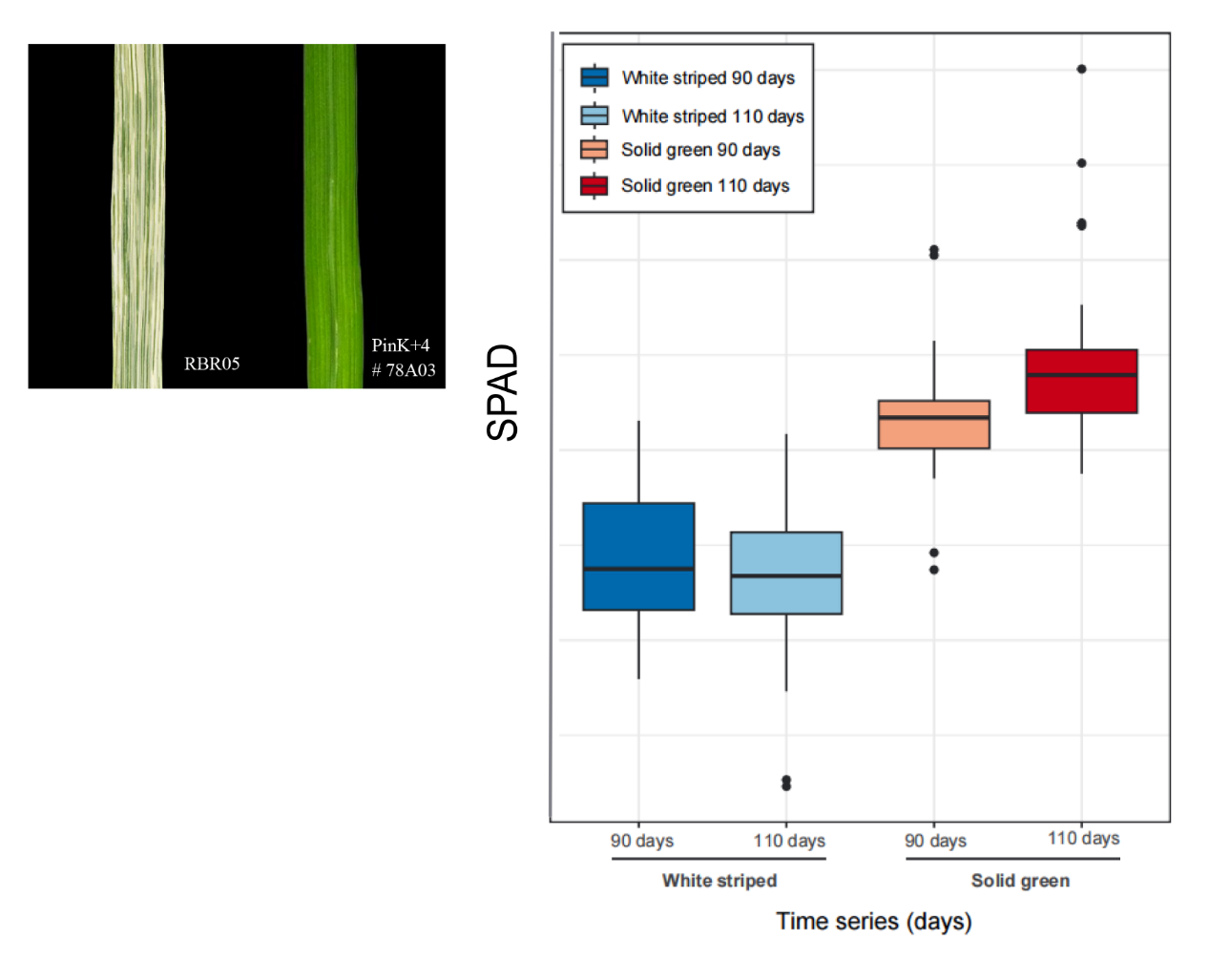


**Supplementary Figure 5:** Leaf phenotype and SPAD chlorophyll readings in RBR05, PinK+4 #78A03, SGL bulk, and *wsl* bulk. Rice leaves with the white-striped trait exhibit alternating green and white sectors along their length compared to solid green leaves. Evaluation of leaf chlorophyll content in RBR05, PinK+4 #78A03, SGL bulk, and *wsl* bulk using a SPAD chlorophyll meter at 90 and 110 days after germination.


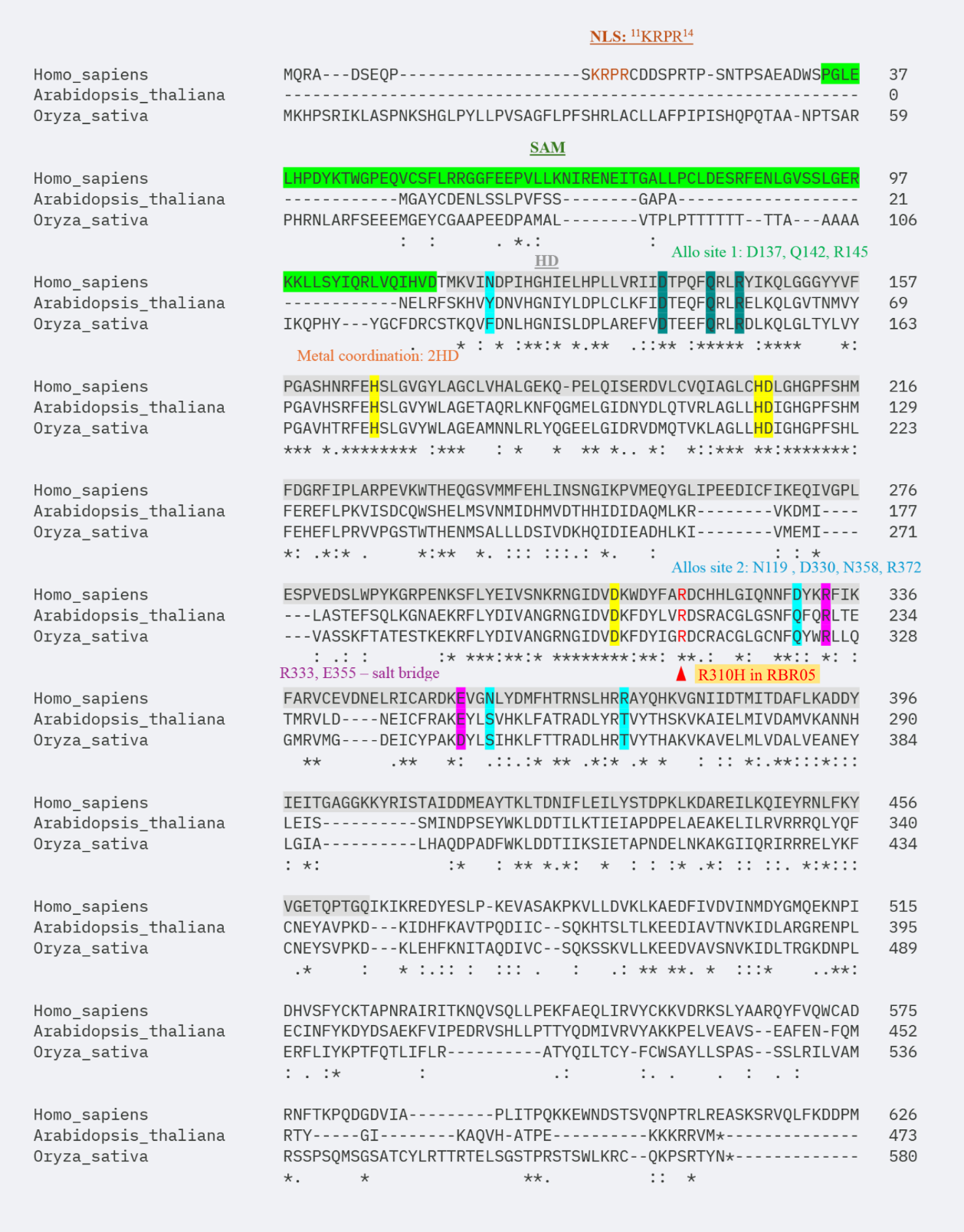


**Supplementary Figure 6:** Multiple sequence alignment of SAMHD1 protein across human (*Homo sapiens*), *Arabidopsis thaliana*, and rice (*Oryza sativa*). The SAM and HD domains in human SAMHD1 are highlighted in green and gray, respectively. Important functional residues in human SAMHD1 are marked. The red triangle indicates the R310H mutation in OsSAMHD1 found in the white-striped leaf RBR05. The alignment was performed using CLUSTAL O (1.2.4) with the following protein accessions: *Homo sapiens* SAMHD1 (NP_056289.2), *Arabidopsis thaliana* VEN4 (AT5G40270.1), and Oryza sativa OsSAMHD1 (LOC_Os01g01920.1).

**Supplementary Table 4:** Evaluation of leaf colour characteristics of white-striped and solid green rice groups by visual assessment and using the SPAD chlorophyll meter to examine the chlorophyll content

**Supplementary Table 5:** Genetic analysis of the *qwsl1_503564* mutant

| Cross set A | Total | SGL | *wsl* | Ratio | Chi-square | *P<0.05* |
| --- | --- | --- | --- | --- | --- | --- |
| PinK+4 #78A03/RBR05_1 | 232 | 172 | 60 | 3:1 | 0.761 | 3.84 |
| PinK+4 #78A03/RBR05_2 | 271 | 211 | 60 | 3:1 | 0.276 | 3.84 |

**Supplementary Table 6:** Summary of whole-genome sequencing data of parental lines, solid green leaf (SGL) and white-striped leaf (*wsl*) bulks

| Sample | Raw Reads (Million) | Cleaned Reads (Million) | Cleaned Base (Gb) | Alignment (%) | Genome  Coverage (%) | Average Depth (×) |
| --- | --- | --- | --- | --- | --- | --- |
| *wsl*-bulk | 2,048.34 | 2,009.62 | 292.55 | 90.27% | 92.71% | 22.85 |
| SGL-bulk | 1,729.12 | 1,692.91 | 245.35 | 91.32% | 92.46% | 19.50 |
| PinK+4 #78A03 | 55.26 | 54.19 | 7.85 | 91.76% | 91.73% | 18.88 |
| RBR05 | 56.79 | 55.75 | 8.09 | 91.03% | 92.05% | 19.26 |

**Supplementary Table 7:** Chromosome-wise distribution of common single nucleotide polymorphisms (SNPs) and Insertions-Deletion (Indels)

| Chr. | Length | Selected SNPs  (Depth ≥ 8) | Selected Indels  (Depth ≥ 8) |
| --- | --- | --- | --- |
| 1 | 43,270,923 | 15,426 | 5,326 |
| 2 | 35,937,250 | 22,101 | 7,536 |
| 3 | 36,413,819 | 19,143 | 5,958 |
| 4 | 35,502,694 | 12,510 | 3,740 |
| 5 | 29,958,434 | 6,656 | 2,430 |
| 6 | 31,248,787 | 8,628 | 2,884 |
| 7 | 29,697,621 | 7,256 | 2,726 |
| 8 | 28,443,022 | 8,507 | 2,842 |
| 9 | 23,012,720 | 4,975 | 1,699 |
| 10 | 23,207,287 | 8,152 | 2,449 |
| 11 | 29,021,106 | 10,313 | 3,293 |
| 12 | 27,531,856 | 9,283 | 3,248 |
| Total |  | 132,950 | 44,131 |
